# The Behavioral Sequelae of Cannabis Use in Healthy People: A Systematic Review

**DOI:** 10.3389/fpsyt.2021.630247

**Published:** 2021-02-16

**Authors:** Maryam Sorkhou, Rachel H. Bedder, Tony P. George

**Affiliations:** ^1^Addictions Division, Centre for Addiction and Mental Health (CAMH), University of Toronto, Toronto, ON, Canada; ^2^Institute of Medical Sciences, University of Toronto, Toronto, ON, Canada; ^3^Department of Psychology, Ryerson University, Toronto, ON, Canada; ^4^Department of Psychiatry, University of Toronto, Toronto, ON, Canada

**Keywords:** cannabis, healthy subjects, cognition, mood, anxiety, psychosis, motivation, intelligence

## Abstract

**Background:** Cannabis is known to have a broad range of effects on behavior, including experiencing a “high” and tranquility/relaxation. However, there are several adverse behavioral sequalae that can arise from cannabis use, depending on frequency of use, potency (e.g., THC content), age of onset, and cumulative exposure. This systematic review examined evidence for cannabis-related adverse behavioral sequalae in otherwise healthy human subjects.

**Methods:** Following PRISMA guidelines, we conducted a systematic review of cross-sectional and longitudinal studies from 1990 to 2020 that identified cannabis-related adverse behavioral outcomes in subjects without psychiatric and medical co-morbidities from PubMed and PsychInfo searches. Key search terms included “cannabis” OR “tetrahydrocannabinol” OR “cannabidiol” OR “marijuana” AND “anxiety” OR “depression” OR “psychosis” OR “schizophrenia” “OR “IQ” OR “memory” OR “attention” OR “impulsivity” OR “cognition” OR “education” OR “occupation”.

**Results:** Our search detected a total of 2,870 studies, from which we extracted 124 relevant studies from the literature on cannabis effects in the non-clinical population. Effects of cannabis on several behavioral sequelae including cognition, motivation, impulsivity, mood, anxiety, psychosis intelligence, and psychosocial functioning were identified. The preponderance of the evidence suggests that frequency of cannabis use, THC (but not CBD) content, age of onset, and cumulative cannabis exposure can all contribute to these adverse outcomes in individuals without a pre-existing medical condition or psychiatric disorder. The strongest evidence for the negative effects of cannabis are for psychosis and psychosocial functioning.

**Conclusions:** Although more research is needed to determine risk factors for development of adverse behavioral sequelae of cannabis use, these findings underline the importance of understanding vulnerability to the adverse effects of cannabis, which has implications for prevention and treatment of problematic cannabis use.

## Introduction

Following nicotine and alcohol, cannabis is the most commonly used psychoactive substance in the world, with a global prevalence of 5.1% in 2016 (1, 2). Cannabis is an illegal substance in most countries but is increasingly becoming a legal drug in various states in the USA, in Portugal and Uruguay, and, as of 2018, nationwide in Canada (3). The trend toward legalization of recreational cannabis use corresponds with heightened acceptance, reduced perception of risk, and an increase in cannabis use among adolescents and adults (4–6).

Cannabis contains over 100 distinct cannabinoids, several of which have demonstrated psychoactive properties (7). Two of the most widely researched cannabinoids are delta-9-tetrahydrocannabinol (THC), and cannabidiol (CBD), which directly modulate the endocannabinoid system in humans. The endocannabinoid system is comprised of at least two cannabinoid receptor types, CB1 and CB2, which are involved in various brain functions, including pain, motivation, memory, mood, and reward processing (8, 9).

THC is the principal psychoactive constituent of the cannabis plant and produces a wide range of transient and dose-dependent effects by acting as an agonist at CB1 receptors (10). In animal models, THC administration reduces anxiety at low doses but increases anxiety at higher doses (11). It also produces transient psychotomimetic effects, including perceptual distortions, paranoia, and euphoria (12). There is evidence that acute administration of THC interferes with numerous behavioral and cognitive processes, including emotional processing, episodic memory, attention, working memory, and reward processing [e.g., (13–15)].

In contrast to THC, CBD has a low affinity for CB1 and CB2 receptors, and its molecular mechanism of action remains poorly understood (7). CBD is thought to inhibit the hydrolysis and reuptake of endocannabinoids and modulate cannabinoid receptors (16, 17) CBD produces markedly different psychological effects in comparison to THC and does not adversely impact cognitive or motor performance during intoxication (18, 19). CBD is devoid of any psychomimetic effects, and both human and animal evidence suggests that CBD has anxiolytic properties (20, 21). Co-administration of CBD and THC may alter the pharmacological effect of THC, such that CBD enhances some of THC's desirable effects while attenuating some of its adverse effects (20, 22–24). For example, a recent systematic review investigating CBD's psychoactive properties, suggested that CBD may offset the psychosis-like effects of THC (25).

Recently, there have been concerns surrounding the increased levels of THC found in present day cannabis, combined with reduced levels of CBD (26). This high-potency cannabis is gaining popularity with recreational users despite a growing body of literature indicating that potent cannabis preparations are associated with adverse health outcomes, including increased risk of psychosis, hypomania, impulsivity, and cannabis use disorder (27–29). Furthermore, with substantial legalization, decreased perceptions of risk associated with cannabis may arise, which may further increase current cannabis use prevalence. For example, nationally representative data from adults across the United States indicated that the perceived risk of recreational cannabis use decreased from 51.3% in 2002 to 40.3% in 2012 (30), even though the THC potency of cannabis has increased from 8.9% in 2008 to 17.1% in 2017 (31). These changes in perception of risk emphasize the need for continued research on the behavioral effects of cannabis use, since there is significant variance concerning the potential harms and benefits of cannabis use.

This systematic review examines experimental, cohort, and cross-sectional studies to determine the effects of cannabis use on behavioral, cognitive, mental health and psychosocial adverse outcomes in non-clinical populations. We sought to address the following aims: (1) to determine the effects of cannabis use on the prevalence and severity of adverse outcomes in people without medical or psychiatric disorders and (2) to determine risk factors associated with the development of adverse outcomes in cannabis users.

## Methods

### Search Strategy

**Figure 1:**
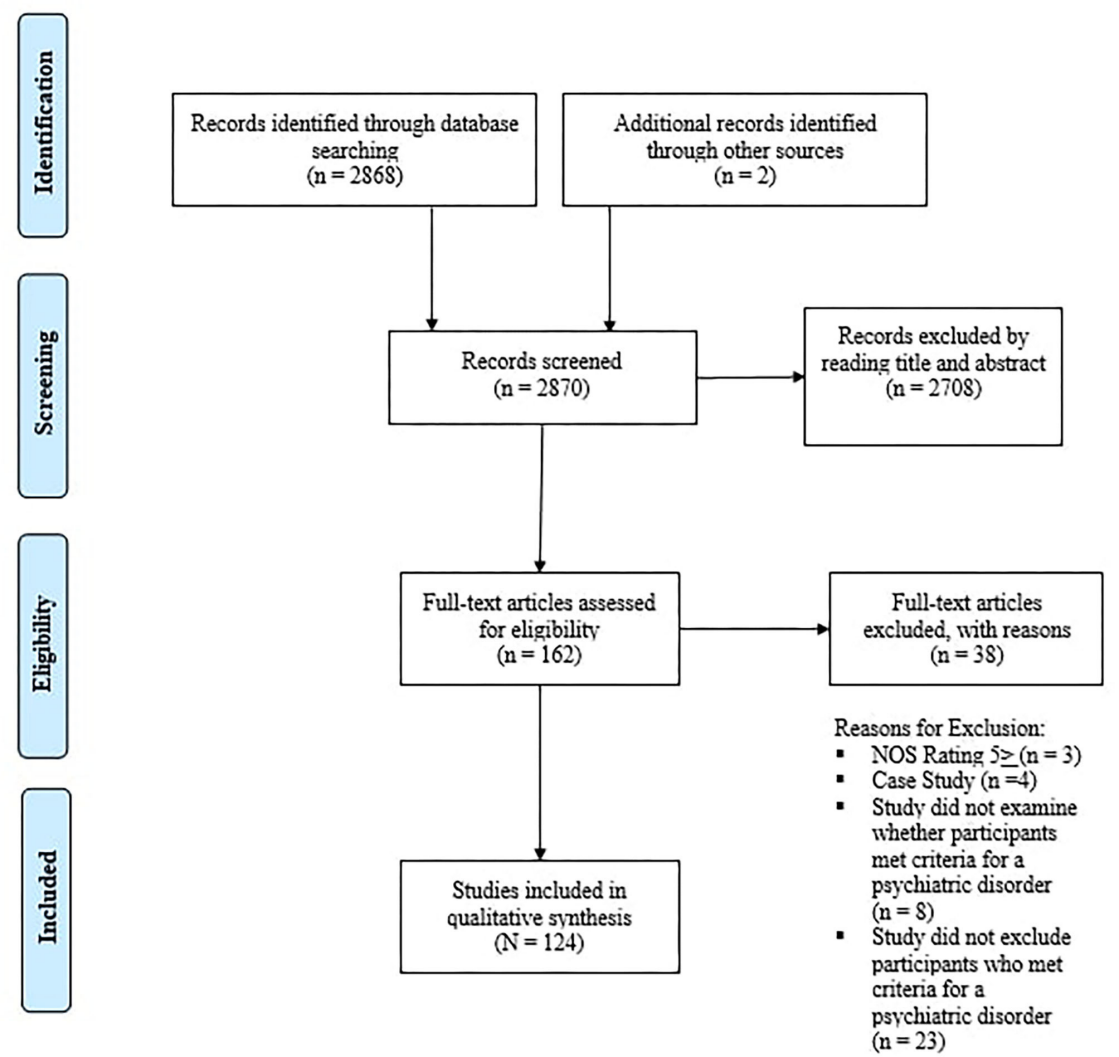
PRISMA Flow Diagram.

Using PubMed and PsycINFO, original, peer-reviewed research articles were searched for based on the PRISMA guidelines by two of the authors (MS and RB) (See Figure 1) (32). Articles available online in the English language between 1990 through the end of October, 2020 were considered. Search terms (found in the title or abstract) utilized to obtain relevant articles were: “cannabis” OR “tetrahydrocannabinol” OR “cannabidiol” OR “marijuana” AND “anxiety” OR “depression” OR “psychosis” OR “schizophrenia” “OR “IQ” OR “memory” OR “attention” OR “impulsivity” OR “cognition” OR “motivation” OR “education” OR “occupation.” Titles and abstracts were screened for relevance by two of the authors (MS and RB), and articles passing this stage were downloaded and assessed for eligibility via full-text review by MS. All uncertainties were assessed and resolved by the senior author (TPG).

The inclusion criteria were: (1) Original studies with experimental, cross-sectional, or cohort designs; (2) studies in which the sample at baseline demonstrated no current medical condition or history of a psychiatric disorder with the exception of cannabis use disorder; (3) studies utilizing validated or objective measures to evaluate cannabis use (e.g., urine toxicology, scores from The Cannabis Use Disorders Identification Test [CUDIT], etc.) (4) studies utilizing validated or objective measures to evaluate the primary outcome (e.g., hospital records, scores from the Beck Depression Inventory, graduation GPA, etc.).

The exclusion criteria were: (1) reviews, meta-analyses, and case studies. Additionally, we employed the Newcastle Ottawa Scale (NOS) to evaluate the quality of eligible cross-sectional and cohort studies in the review (33). The NOS allows a maximum score of nine and evaluates studies on three broad domains; (1) the selection of the study groups; (2) the comparability of the groups; and (3) the verification of the outcome of interest. Studies receiving a score of 5 or lower on the NOS indicated a high risk of bias, and consequently were excluded from the review (See Supplementary Tables 2,3 for the quality ratings of the included and excluded studies, respectively).

### Evidence Ratings

We recorded the following variables from each study: author, publication year, study design, sample size, study population, follow-up time, outcome measures, level of cannabis use, matched variables, and relevant findings (see Supplementary Table 1 for further information). To determine whether there is a dose-dependent relationship between cannabis and the primary outcomes, we adapted a classification system utilized by Batalla et al. (34) which evaluated levels of cannabis use. We classified both cross-sectional and experimental designs. Cannabis dependent persons are those who meet the criteria for cannabis use disorder on the DSM-5 or DSM-IV at the time of the study. Chronic cannabis users are persons who do not meet the DSM-5 or DSM-IV criteria for cannabis use disorder but use cannabis 3+ times a week for at least 1 year. Recreational cannabis users are persons who use cannabis between one and four times a month, and controls were persons use had used cannabis <10 times in their lifetime.

To determine whether the accumulated evidence implicates a neutral or negative effect of cannabis for each domain, we calculated the proportion of studies evincing a negative effect of cannabis use against the total number of studies (See Table 2 for a summary of the level of evidence ratings) as follows:

1 = 0–19% corresponds with strong evidence of no effects of cannabis use
2 = 20–39% corresponds with moderate evidence of no effects of cannabis use
3 = 40–59% corresponds with mixed evidence of neutral or negative effects of cannabis use
4 = 60–79% corresponds with moderate evidence of negative effects of cannabis use
5 = 80–100% corresponds with strong evidence of negative effects of cannabis use.

## Results

We identified 2,870 hits of which 159 were considered potentially relevant, based on title and abstract inspection (See Figure 1 for methods overview). Detailed examination of the potentially relevant publications reduced the sample to 127 studies that were within the inclusion criteria set for this systematic review. However, after examining the quality of eligible studies via the NOS, three studies were excluded from the review. Overall, 124 studies were included in the review with publication dates ranging from 1995 to 2020. Studies were conducted in a broad range of countries, and comprised of 48 cohort designs, 26 placebo-controlled, counter-balanced, experimental designs, and 50 cross-sectional designs (see Table 1).

**Table 1:** Review of evidence for acute and chronic effects of cannabis use on behavioral outcomes.

**Behavioral Outcome Measured**	**Negative Effects**	**References**	**Positive or Neutral Effects**	**References**	**Conclusions**
Verbal, Episodic, and Working Memory (*N* = 37)	There is evidence that acute and chronic cannabis use beginning in adolescence is associated with impairments in working memory, episodic memory, and verbal learning	(13, 24, 35–56)	Other evidence suggests that cannabis use is not associated with impairments in episodic memory, verbal working, or verbal learning	(14, 35, 56–66)	Overall, there is a moderate level of evidence implicating a negative relationship between cannabis use and verbal, working, or episodic memory. A total of 24/37 (64.9%) of included studies assessing these behavioral sequelae observed a negative effect of cannabis use
Visuospatial Memory (*N* = 6)	Only one experimental study found that THC administration corresponds with impairment in visuospatial memory	(41)	Most evidence suggests that cannabis use is not associated with impairments in visuospatial memory or visuospatial working memory	(40, 66–70)	There is little evidence implicating a relationship between cannabis use and impairments in visuospatial memory. Only 1/6 (16.7%) of included studies assessing this behavioral sequela observed a negative effect of cannabis use
Attention (*N* = 20)	There is evidence that chronic cannabis use is associated with impairments in divided attention and sustained attention	(36, 40, 53, 64, 70–77)	Other evidence that chronic cannabis use is not associated with impairments in selective attention	(35, 44, 48, 57, 62, 66, 78, 79)	Overall, there is a moderate level of evidence implicating a negative relationship between cannabis use and attention. 12/20 (60%) of included studies assessing this behavioral sequela observed a negative effect of cannabis use
Processing Speed (*N* = 6)	There is evidence that cannabis use and acute THC intoxication is associated with impairments in information processing	(36, 64, 72)	There is other evidence that chronic cannabis use does not lead to impairments in information processing	(56, 63, 65)	Overall, there is a mixed level of evidence implicating a negative relationship between cannabis use and processing speed. 3/6 (50%) of included studies assessing this behavioral sequela observed a negative effect of cannabis use
Executive Function (*N* = 20)	There is evidence from multiple study designs that cannabis use is associated with impairments in executive functioning, decision-making, and planning	(35, 47, 49, 74, 77, 80–86)	There is other evidence that chronic cannabis use does not impair executive functioning	(44, 48, 62, 65, 66, 87–89)	There is a moderate level of evidence implicating a negative relationship between cannabis use and executive function. 12/20 (60%) of included studies assessing this behavioral sequela observed a negative effect of cannabis use
Impulsivity/Inhibitory Control (*N* = 17)	There is evidence that acute THC intoxication and cannabis use beginning in adolescence is associated with greater impulsivity or impairments in inhibitory control	(41, 44, 48, 63, 71, 74, 90–93)	However, some studies assessing acute THC intoxication or chronic cannabis use in adults is not associated with greater impulsivity or impairments in inhibitory control	(35, 38, 56, 70, 94–96)	There is a mixed level of evidence implicating a negative relationship between cannabis use and inhibitory control. 10/17 (58.8%) of included studies assessing this behavioral sequela observed a negative effect of cannabis use
Intelligence (IQ) (*N* = 7)	There is some evidence that cannabis use beginning in adolescence is correlated with a minor decrease (1–2 points) in IQ in adulthood	(47, 55, 86)	Other evidence suggest that chronic cannabis use does not impact global IQ in adulthood after adjusting for potential confounds	(42, 97–99)	There is a mixed level of evidence implicating a negative relationship between cannabis use and intelligence. 3/7 (42.7%) of included studies assessing this behavioral sequela observed a negative effect of cannabis use
Motivation (*N* = 6)	There is evidence supporting the view that chronic cannabis users demonstrate amotivation and reduced reward processing than non-users	(100–103)	Two case-control studies found that cannabis use is not associated with impairments in motivation	(104, 105)	There is a moderate level of evidence implicating a negative relationship between cannabis use and motivation. 4/6 (66.7%) of included studies assessing this behavioral sequela observed a negative effect of cannabis use
Psychosocial Functioning (*N* = 8)	There is substantial evidence that daily or weekly cannabis use throughout high school is associated with lower educational and occupational attainment	(106–112)	One study indicated that cannabis use in high school is not associated with educational performance	(98)	There is a strong level of evidence implicating a negative relationship between cannabis use and psychosocial functioning. 7/8 (87.5%) of included studies assessing this behavioral sequela observed a negative effect of cannabis use
Depression (*N* = 27)	There is evidence that daily or weekly cannabis use beginning in adolescence is a risk factor for a diagnosis of major depressive disorder (MDD) in adulthood	(54, 90, 100, 107, 109, 113–123)	Some evidence from case-control designs suggest that cannabis use is not associated with depression and acute administration of THC may decrease depressive symptoms for a short period of time	(51, 61, 124–132)	There is a mixed level of evidence implicating a relationship between cannabis use and increased depression. 16/27 (59.3%) of included studies assessing this behavioral sequela observed a negative effect of cannabis use
Anxiety (*N* = 23)	There is evidence that chronic cannabis use beginning in adolescence and acute, high dose administration of THC is associated with an increase in anxiety symptomology	(13, 14, 41, 52–54, 90, 100, 107, 116, 119–121, 133)	However, there is also evidence that acute, low dosing of CBD is associated with a decrease in anxiety symptomology	(61, 113, 115, 122, 127, 129, 134–136)	There is a moderate level of evidence implicating a relationship between cannabis use and increased anxiety. 14/23 (60.9%) of included studies assessing this behavioral sequela observed a negative effect of cannabis use
Psychosis (*N* = 27)	There is substantial evidence that chronic cannabis use in adolescence and acute, high dose administration of THC is associated with an increased risk for a psychotic disorder or acute psychosis, respectively	(13–15, 24, 53, 54, 93, 107, 124, 132, 137–152)	There is minimal evidence that cannabis is not associated with greater psychotic symptoms	(61, 153)	There is a strong level of evidence implicating a relationship between cannabis use and increased risk for psychosis. 25/27 (92.6%) of included studies assessing this behavioral sequela observed a negative effect of cannabis use

### Cognition

#### Memory

Four longitudinal studies evaluating the impact of cannabis use and memory were included (35, 42, 47, 63). Becker et al. (35) investigated differences among multiple cognitive domains within daily, adolescent, cannabis users and non-users at baseline and 2 years later. At follow-up, cannabis users demonstrated significant impairments in working memory and verbal learning relative to non-users. Moreover, an earlier age of cannabis use onset corresponded with more severe impairments at follow-up. Similarly, following a birth cohort of 1,037 individuals, Meier et al. (47) assessed neurocognitive functioning in participants at ages 13 and 38, while assessing cannabis use at ages 18, 21, 26, 32, and 38. Persistent cannabis use beginning in adolescence corresponded to impairments in working memory and verbal learning, in addition to other neurocognitive domains. However, a recent 14 year longitudinal study following adolescents annually, found no relationship between cannabis or alcohol use and performance in tasks evaluating verbal memory (63). Moreover, in a study comparing the effects of extended (28 day) of cannabis abstinence and reinstatement of cannabis use in people with schizophrenia vs. healthy controls with cannabis use disorder, there were no significant effects of 28 days of cannabis abstinence or reinstatement on verbal learning, verbal memory (% retention on HVLT-R task), and visuospatial memory in healthy controls, as compared to significant (>40%) improvements in verbal learning and memory with abstinence and impairment with cannabis use reinstatement in schizophrenia patients (62).

Twelve cross-sectional designs found no significant impairments in memory among cannabis users (24, 56–60, 64–69). Three of these studies investigated visuospatial working memory and independently employed fMRI to investigate potential differences in brain activity (67–69). Interestingly, although the three studies failed to suggest a behavioral deficit in cannabis users, the researchers all found unusual brain activity in cannabis users while performing the visuospatial memory task. Additionally, four other cross-sectional designs concluded no significant impairments among cannabis users in working memory (57–60). An fMRI study assessing working memory among a small sample of abstinent but frequent cannabis-using adolescents obtained no behavioral impairments on the task (60). However, during the task, cannabis users demonstrated increased brain activity in regions implicated in working memory, suggesting a reliance on neural compensatory strategies. These neurofunctional results were obtained in a similar study which found neural differences between cannabis users and non-users on a Sternberg-type working memory task, but no behavioral differences (57). In another study comparing adult, chronic cannabis users and controls on tests evaluating visuospatial memory, verbal memory, and executive functioning over a 28 day period, cannabis users demonstrated significant deficits in verbal memory during the first week of abstinence, but by Day 28, any significant differences between groups diminished (66). Moreover, no relationship between cannabis use frequency and performance at Day 28 emerged, presenting evidence for re-instatement of cognitive abilities with abstinence.

In contrast, 11 cross-sectional designs concluded that cannabis use is associated with memory impairments (24, 36, 39, 40, 43–46, 48, 49, 56). Morgan et al. (24) investigated whether the type of cannabinoid that users consumed led to differing impairments in memory. Splitting cannabis users into high THC/high CBD and high THC/low CBD groups, individuals who smoked high THC/high CBD cannabis did not demonstrate any deficits in immediate or delayed recall on an episodic memory task (24). However, high THC/low CBD users performed significantly worse on the task assessing episodic memory, leading to the authors' proposition that CBD may attenuate the harmful effects of THC on cognition. Additionally, three studies comparing 2–4 week cannabis abstinent users and non-users on measures of verbal learning and memory indicated that despite abstinence, users performed significantly worse than non-users (40, 44, 49). However, one of these studies found that among a 21 day cannabis abstinence intervention, slight improvements in verbal memory were obtained in cannabis users between Weeks 2 and 3, indicating partial recovery of neurocognitive functioning (40).

Similarly, the majority of experimental studies in which healthy volunteers received varying doses of THC suggest a negative impact of cannabis use on verbal learning, episodic memory, and working memory (13, 37, 41, 50, 51, 53). A recent study examined acute and delayed effects of THC intoxication on susceptibility to false memory among occasional cannabis users (50). Intoxicated participants generated significantly more spontaneous and suggestion-based false memories immediately after intoxication in comparison to placebo. The authors surmise that THC-intoxicated individuals may demonstrate a tendency toward more liberal responding to memory-related questions due to reductions in alertness and impaired abilities in forming learning associations. Additionally, the effects were most prominent at immediate in comparison to delayed recall. One experimental design administering vaporized THC (12%) or placebo to male, recreational, adolescent and adult cannabis users found that in comparison to adolescent users, adults exhibited greater impairments in a spatial working memory task following intoxication (41).

Three experimental designs found no relationship between acute cannabinoid intoxication and memory impairments (14, 61, 70). Bhattacharyya et al. (14) conducted an fMRI studying neurocognitive function during the Hopkins Verbal Learning Task after acute oral THC administration (10 mg) to healthy, occasional cannabis users. Although overall task performance was unaffected, THC attenuated brain activation in regions associated with episodic memory. These findings implicate that greater neural effort is required after THC administration to maintain normal levels of task performance. Additionally, Englund et al. (61) investigated whether a pre-treatment of 10 mg of oral Δ-9-tetrahydrocannabivarin (THCV) prior to 1 mg of THC intoxication would lead to cognitive and clinical effects among a non-clinical sample of men. The authors found that pre-treatment with THCV protected participants from memory impairments in a delayed memory recall task.

Overall, the literature suggests that acute THC intoxication produces acute impairments in verbal learning, episodic, and working memory. Moreover, the literature suggests a dose-dependent relationship between levels of cannabis use and long-term impairments within this cognitive domain.

#### Attention

Seven cross-sectional studies found a significant association between cannabis use and attention deficits (36, 40, 46, 64, 73, 74, 77). Fontes et al. (74) compared a sample of adult early-onset (before age 15), late-onset (after age 15), and non-users on a sustained attention task. Results indicated that early-onset users demonstrated significant impairments in comparison to controls and late-onset users. Similar findings were also obtained by Jacobsen et al. (73), where adolescent cannabis users performed significantly worse on a sustained attention task relative to controls and adolescent tobacco users. However, five cross-sectional designs indicated no differences in attention between cannabis users and non-users (44, 48, 57, 66, 78). Fridberg et al. (78) and Jager et al. (57) each investigated whether chronic cannabis users demonstrated impairments in selective attention, both in terms of behavioral performance and abnormal brain activity. Although both studies found that cannabis users performed equally fast and accurate as controls, cannabis users demonstrated abnormal brain activity as evaluated through fMRI and EEG, respectively.

Five experimental designs found that shortly after THC intoxication, adult participants without a medical or psychiatric disorder demonstrated significant deficits in attention (53, 70–72, 76). Desrosiers et al. (70) found that after acute, THC intoxication, recreational cannabis users performed significantly worse on a divided attention task in comparison to chronic users. The researchers surmised that the differences in performance are attributed to tolerance development in frequent smokers. Two major limitations of this study should be noted, however. First, performance while sober was not obtained in the study, so conclusions on overall attention impairments were limited. Additionally, occasional and frequent users' performance was not compared against a group of healthy controls to further evaluate the significance of their impairments. A more recent experimental design compared the effects of THC-dominant and THC/CBD equivalent cannabis ratios among a non-clinical sample on a task of divided attention (72). Unlike prior research (15, 24), the co-administration of CBD did not mitigate any adverse, cognitive effects of THC, and participants demonstrated significant attention deficits in both conditions. However, one experimental design administering either 0.015 or 0.03 mg/kg of THC to former, recreational cannabis users without a medical or psychiatric disorder found no impairments in sustained attention, as measured via the Rapid Visual Processing Task (79).

One longitudinal study following adolescents for 2 years found no relationship between cannabis use and speeded attention (35). However, users did demonstrate impairments in other cognitive domains, including working memory and planning. Rabin et al. (62) compared the effects of extended (28 day) cannabis abstinence and reinstatement of cannabis use in people with schizophrenia and healthy controls with cannabis use disorder and found no improvements in sustained attention among both groups of abstainers. Improvements were not obtained in multiple cognitive domains as well, including executive function and visuospatial working memory.

Overall, the literature suggests a relationship between chronic cannabis use and impairments in attention. However, more evidence is required to ascertain whether there is a dose-dependent effect of levels of cannabis use and deficits in this cognitive domain. Additionally, acute THC-intoxication appears to produce temporary impairments in attention.

#### Processing Speed

Two cross-sectional studies and one experimental study found a relationship between cannabis use and impairments in processing speed (36, 64, 72). Thames et al. (36) evaluated whether cannabis use in the previous month impacted neurocognitive functioning in a non-clinical adult sample. Participants were classified as either recent users (last use within 4 weeks since study), remote cannabis users (last use >4 weeks since study), and non-users (no report of cannabis use). Recent users demonstrated impairments in information processing speed in addition to other cognitive domains (e.g., attention, working memory, and executive functioning) in comparison to past- and non-users (36). Interestingly, past users did not differ from non-users in any domain except for executive functioning, suggesting that cannabis abstinence can restore previous cognitive abilities. A rigorously controlled experimental design compared whether high THC/CBD vs. equivalent THC/CBD cannabis ratios would produce differential cognitive impairments among volunteers with no history of a psychiatric disorder (72). In an information processing speed task, 1:1 THC/CBD intoxication produced greater impairments in participants in comparison to the high THC/CBD condition, suggesting that CBD may not effectively prevent cognitive impairments associated with THC intoxication.

However, one recent 14 year longitudinal study following adolescents at baseline yearly, determined no relationship between cannabis or alcohol use and performance in tasks evaluating processing speed (63). Furthermore, two cross-sectional designs evaluating processing speed among older adults without a current medical condition or psychiatric disorder, no relationship between cannabis use and this cognitive domain emerged (56, 65). Burggren et al. (65) evaluated verbal memory, processing speed, and executive functioning among a sample of older adults who were former, chronic, cannabis users in addition to non-users and found that while former users performed worse than non-users on all cognitive domains, the differences were not significant. Similarly, Thayer et al. (56) obtained no behavioral differences in processing speed and other cognitive domains across a sample of older adults who were either current, chronic, cannabis users or had no history of cannabis use.

Overall, the evidence remains mixed on whether cannabis use alters processing speed, and whether there is a dose-dependent relationship between cannabis use and impairments in this neurocognitive ability.

#### Executive Function

Three longitudinal designs implicate a significant relationship between adolescent cannabis use and impairment in executive functioning (35, 47, 86). Castellanos et al. (86) concluded that frequent cannabis use among adolescent boys was associated with a significant decline in executive functioning by the end of high school. The relationship persisted even after controlling for high school graduation and other substance use. Additionally, a more recent longitudinal study obtained similar findings. Becker et al. (35) found that in comparison to adults without a history of cannabis use, chronic cannabis users who began use in adolescence, demonstrated significant reductions in planning and verbal learning at a 2 year follow-up. Additionally, reducing cannabis use did not ameliorate users' cognitive deficits.

Eight cross-sectional designs obtained a significant relationship between cannabis use and impairments in executive functioning (49, 74, 77, 80–83, 85). Four of these studies implicated that chronic cannabis users demonstrate significant impairments in decision-making in comparison to their non-using counterparts (77, 80, 81, 85). Two studies comparing early-onset (use began before age 16), late-onset (use began at or past age 16), and non-using controls in tasks evaluating executive functioning (e.g., Stroop task, Frontal Assessment Battery, and Wisconsin Card Sorting Test) suggested that only early-onset users performed poorly on these tests in comparison to both the late-onset users and healthy controls (74, 82).

However, seven cross-sectional studies found no deficits between cannabis users and non-users on tasks assessing executive functioning (48, 56, 65, 66, 87–89). In a study comparing numerous cognitive abilities between controls and older adults who were currently using cannabis, no differences between groups on any neuropsychological test emerged (56). Furthermore, employing structural MRI, the authors also obtained no significant differences between groups in white or gray matter density. However, one limitation of this study is that duration and levels of use was not specified, which may have impacted the findings. Moreover, employing fMRI, Hatchard et al. (88) obtained no performance differences between cannabis users and non-users on a Counting Stroop Task. However, in contrast to non-users, cannabis users displayed more intensive and extensive BOLD responses. The researchers surmise that the recruitment of additional brain regions among cannabis users may be a neural compensatory strategy to maintain their behavioral performance. Additionally, in one study comparing 25 day abstinent, adult, chronic cannabis users, cocaine users, and non-using controls on the Iowa Gambling Task, cocaine users demonstrated significant learning impairments in comparison to cannabis users and controls (89). Although abstinent-cannabis users did not significantly differ from controls on the task, their performance was consistently lower. This finding implicates that cannabis use may negatively impact executive functioning, but with abstinence, cognitive deficits are somewhat reversible. Finally, the researchers also obtained a dose-dependent effect of cannabis use on IGT performance, which implicates that this substance may affect cognition.

Overall, our review obtained a moderate level evidence concerning the effects of cannabis use and impairments in executive functioning. However, levels of cannabis use did not consistently correspond with greater impairments in this neurocognitive domain.

#### Impulsivity/Inhibitory Control

Only one longitudinal study investigated the role of cannabis use on the course on inhibitory control (63). Following adolescents between the ages of 12–15 annually, for 14 years, Infante et al. (63) found that greater cumulative cannabis use in adolescence was associated with deficits in inhibitory control in adulthood. The effects remained after controlling for relevant confounders including alcohol use.

Concerning cross-sectional designs, six studies also concluded that cannabis users exhibit greater impulsivity and poorer inhibitory control than non-users (44, 48, 64, 74, 91, 92). Lisdahl and Price (64) examined whether past-year cannabis use among adolescents and young adults corresponded with impairments in inhibitory control. After controlling for numerous confounders, cannabis use corresponded with poorer cognitive inhibition in a dose-dependent fashion. Additionally, there is some evidence that cannabis use corresponds with greater impulsivity in daily life. Ansell et al. (91) employed Ecological Momentary Assessment (EMA) to examine more immediate effects of cannabis on same day and subsequent day reports of impulsivity among a sample of chronic, cannabis-using adults. The authors found that independent of alcohol consumption, cannabis use was associated with same day increases in impulsivity and predicted next day increases in impulsivity.

However, three separate cross-sectional studies reported no impulsivity differences between cannabis users and non-users (38, 94, 95). In a study comparing inhibitory control among 28 day abstinent adolescent cannabis users and non-users, no significant differences between the groups' performance on a go/no-go task emerged (95). Similar findings were obtained by a more recent study, where regular, young-adult cannabis users and matched non-users performed similarly on an inhibitory task (94). However, when the authors compared early-onset (prior to age 16) and late-onset (ages 16 or later) cannabis users, individuals in the former group made greater errors of commission, but the results remained insignificant.

Although the literature is ambiguous on whether cannabis use produces long-term effects on impulsivity, there is evidence that acute administration of THC produces impairments in inhibitory control. Five experimental designs suggest a relationship between THC intoxication and increased impulsivity (41, 71, 76, 90, 93). Employing a double-blind, placebo-controlled design, Ramaekers et al. (71) compared the effects of 500 μg/kg of THC among occasional and chronic cannabis-using adults in the stop signal task (SST) 35 min, 3 h 30 min, 5 h 30 min, and 7 h 30 min post-THC administration. Both occasional and heavy cannabis users demonstrated impaired inhibitory control in the intoxicated condition, where worst performance arose 35 min after intoxication. To replicate this study's findings, Theunissen et al. (76) repeated the former study with a different group of occasional and chronic cannabis users and found that both users demonstrated significant impairments in the SST 45 min after THC administration.

Overall, whether chronic cannabis use reduces inhibitory control in a dose-dependent manner is less clear. However, the literature suggests that acute administration of THC leads to greater impulsivity and poorer inhibitory control.

### Intelligence (IQ)

Six longitudinal studies assessing the impact of cannabis use on IQ were included (42, 47, 86, 97–99). Two cohort studies concluded that there was a significant association between cannabis use beginning in adolescence and a decline in IQ (47, 86). Castellanos-Ryan et al. (86) concluded that frequent cannabis use among adolescent boys is associated with a significant decline in verbal IQ scores by the end of high school. Moreover, the authors found that poor short-term and working memory in pre-adolescence was associated with an earlier age of onset for cannabis use. Despite these findings, the relationship between cannabis use and verbal IQ was mediated by a reduction in high-school graduation rates. Meier et al. (47) conducted a large, national representative, birth-cohort design (*N* = 1,748) which explored the relationship between cannabis use from preadolescence into adulthood and potential changes in IQ and cognitive abilities. Individuals who received a diagnosis of cannabis use disorder before the age of 18 demonstrated an eight-point decline in IQ by the age of 38 in comparison to peers who never used cannabis or began use after the age of 18 (47). This relationship persisted after statistically adjusting for alcohol, tobacco and other drug use, schizophrenia, and educational level.

However, four cohort designs assessing cannabis use and IQ changes indicate that there is no direct relationship between these two variables (42, 97–99). In a birth-cohort twin study, adolescent cannabis use was associated with a decline in IQ and impaired executive functioning, but twins who used cannabis performed no worse than their co-twin without a history of cannabis use (42). The authors subsequently suggest that family background factors contribute to a spurious relationship between cannabis use and impaired executive functioning in the general population. An additional, cohort, twin design also indicated no relationship between cannabis use and IQ decline (99). Participants' IQ was measured prior to cannabis use between the ages of 9 to 12 years old, and again 8 years later. Cannabis use between this period corresponded with reduced scores in IQ at follow-up, however no clear relationship between frequency of use and IQ emerged. Consequently, the authors determined that the declines in IQ reflected the effects of familial or genetic factors that predated cannabis use onset. In a separate study investigating cognition and verbal IQ among 28 day abstinent early-onset (smoking before age 17) cannabis users, late-onset (smoking at or after age 17) cannabis users, and controls, only early-onset users performed poorly on measures evaluating verbal IQ (55). However, this relationship did not persist after controlling for relevant variables, including familial and childhood factors.

Currently, the literature remains mixed on whether chronic cannabis use impacts intelligence and IQ. Some studies suggest that there is a dose-dependent relationship between cannabis use and IQ scores, while other evidence suggests that there is no relationship between these variables.

### Motivation

Four cross-sectional studies included in the review implicate that cannabis users demonstrate motivation impairments in comparison to non-users (100–103). In a study examining the influence of reward on mood and performance on the spatial delayed response task in an adult sample of chronic cannabis users, tobacco smokers, and non-smoking controls, cannabis users rated their mood as significantly lower than smokers and controls during the reward conditions (101). The authors concluded that cannabis use may reduce reward processing at a behavioral level. Additionally, Lane et al. (103) found that on a monetary task assessing perseverative responding, adolescent cannabis users switched to the non-work, but less rewarding task significantly earlier than non-users.

However, two cross-sectional studies obtained no relationship between cannabis use and reductions in motivation (104, 105). In one study comparing adolescent cannabis users and non-users on a self-report battery examining high school students' motivation, no differences emerged (105). Additionally, Jager et al. (104) compared the performance of abstinent cannabis-using boys and non-using boys on the monetary incentive delay (MID) task, which assesses motivation and reward processing. Although no behavioral differences between groups emerged, a significant limitation of the study should be noted. Cannabis abstinence was not controlled for and varied from 1 to 16 weeks, which may have impacted the observed findings. Overall, the evidence suggests a dose-dependent relationship between cannabis use and impairments in motivation.

### Psychosocial Functioning

We identified eight longitudinal studies demonstrating that cannabis use has adverse effects on psychosocial functioning, including occupational and educational attainment (86, 106–112). Follow-up times ranged from 1 to 35 years. One study using a large, nationally representative sample found that among students from grade 9–12, individuals who used cannabis at baseline were less likely to attend class, complete their homework, and obtain or value high grades relative to their abstaining peers at year 2 and 3 (111). Furthermore, frequent cannabis use reduced the likelihood of planning to pursue either a graduate or professional degree post-graduation. A recent birth cohort study assessed four trajectories of cannabis use, including non-users, adolescent-limited, adult-onset, and chronic-adolescent users (107). Individuals who began cannabis use in adolescence and continued use throughout adulthood demonstrated the worst psychosocial functioning at age 35, while the non-user group reported the highest level of well-being. Moreover, heavy cannabis use in adolescence corresponded with an increased risk of adverse outcomes at ages 30–35, including a reduced likelihood to attain a postsecondary degree, a lower weekly income, a greater likelihood to rely on welfare, a greater likelihood of being unemployed, and a greater likelihood of being arrested. In contrast, one prospective, cohort design concluded that although there was a significant relationship between cannabis use at age 15 and educational performance at age 16, this relationship became non-significant after adjusting for relevant variables such as cigarette smoking, childhood conduct problems, and childhood depressive symptoms (98).

Overall, the evidence obtained in this review implicates a dose-dependent relationship between levels of cannabis use and poorer psychosocial outcomes.

### Depression

We identified two experimental studies demonstrating a negative effect of THC in mood among a group of adults without a history of medical or psychiatric illness (15, 122). In one study, male participants received 10 mg of THC and reported their mood 1, 2, and 3 h post- THC administration. Relative to placebo, participants reported significantly elevated levels of dysphoria (15). However, two separate experimental studies administering THC to a non-clinical sample did not obtain similar findings (51, 61). Instead, both studies found that there was a dose-dependent relationship between THC consumption and pleasurable mood ratings.

Concerning longitudinal findings, we identified thirteen studies that suggested a relationship between cannabis use and a greater likelihood of developing major depression (107, 109, 113–121, 123, 131). However, six of these studies specifically outlined that only heavy or chronic (<4 times/week) adolescent cannabis use is a risk factor for depression in adulthood. Otten and Engels (123) investigated the relationship between cannabis use, depression, and the serotonin transporter gene (5-HTTLPR). The serotonin transporter gene is considered a significant candidate gene for its role in depression [for a review, see (154)]. Specifically, this gene encodes the serotonin transporter protein, which is responsible for the reuptake of serotonin from the synaptic cleft into the presynaptic neuron. The authors identified that cannabis use increases the risk for an increase in depressive symptoms over a 5 year period but only in users with the short allele of the 5-HTTLPR genotype. One study also investigated whether sex differences emerged when evaluating the effect of adolescent cannabis use and depressive symptomology in young adulthood (121). A state-wide secondary school sample of 1,601 students aged 14–15 were followed for 6 years. Daily use in female adolescents was associated with a 5-fold increase in the odds of reporting depression and anxiety after adjustment for concurrent use of other substances. Weekly or more frequent cannabis use in adolescents predicted an ~2-fold increase in risk for later depression and anxiety at follow-up, even after adjusting for potential confounders.

However, 10 separate cohort designs suggest that cannabis use is not associated with an increased risk of a future depression diagnosis (124–132, 155). Despina et al. (125) assessed 1,606 adolescents and obtained data on frequency of cannabis use and serious suicidal ideation at ages 15, 17, and 20 years. While cannabis use did not predict depressive symptomology or suicide ideation, depression predicted subsequent cannabis use, even after adjusting for possible confounders, including other substance use. Additionally, it is important to note that five of these longitudinal designs only considered adult cannabis use at baseline, which limited conclusions concerning the causal relationship between adolescent cannabis use and subsequent depressive symptomology (124, 126, 127, 131, 155).

Three cross-sectional designs also concluded that relative to non-users, cannabis users had more severe depressive symptomology (54, 100, 102). Employing positron emission tomography (PET), Bloomfield et al. (102) evaluated the relationship between dopaminergic function and subjective apathy in a sample of adult, chronic cannabis users. In comparison to normative data from adult without a history of cannabis use, cannabis users reported significantly greater levels of apathy and demonstrated reduced dopamine synthesis capacity.

Overall, the evidence obtained is mixed regarding the impact of cannabis use on depressive symptomology. Some studies suggest a dose-dependent relationship between levels of cannabis use and increased risk of depression, while other evidence found no relationship between these variables after controlling for relevant confounds.

### Anxiety

Three prospective cohort designs implicate cannabis use as a significant risk factor for subclinical anxiety symptomology (116, 119, 133). One longitudinal study determined that among adolescent boys, increases in past-year cannabis use corresponded with increases in depressive and anxious symptomology the following year (119). Similarly, Hayatbakhsh et al. (116) found that after controlling for numerous confounding variables, cannabis use before the age of 15 correlated with greater anxious symptomology at age 21 among a national representative sample of adolescents (*N* = 3,239).

Four prospective designs suggest chronic cannabis use as a significant risk factor for the development of an anxiety disorder (107, 120, 121, 133). A birth-cohort study collecting information until participants reached 21 years old determined that frequent cannabis use in adolescence predicted a more than 2-fold increase in the diagnosis of an anxiety disorder by the final follow-up (133). This relationship persisted after controlling for tobacco, alcohol, and other substance use. Additionally, anxiety symptomology never predicted subsequent cannabis use. An additional birth-cohort longitudinal design investigated the impact of cannabis use and internalizing problems until age 35 (107). Adolescent-onset cannabis users and young-adulthood cannabis users were significantly more likely to be diagnosed with an anxiety disorder in comparison to adolescent-limited cannabis users, and non-users. The effect persisted even after controlling for childhood, other substance use, and familial factors. Additionally, one cohort study following a group of 14 year-olds for 15 years obtained no consistent relationship between adolescent cannabis use and a diagnosis of major depressive disorder at 29, but daily cannabis use in adolescence was a significant risk factor for development of generalized anxiety disorder at 29, even after adjusting for baseline confounders and other concurrent drug use (120). The researchers also found that overall, among participants, there was a reduction in cannabis use over young adulthood. However, among individuals who developed an anxiety disorder, the pattern of use was associated with either the maintenance or increasingly frequent use of cannabis throughout young adulthood. Despite these findings, six prospective studies obtained no relationship between cannabis use and an increased risk for a diagnosis of an anxiety disorder or an increase in subclinical anxiety symptomology (113, 115, 127, 129, 135, 155). One UK birth cohort study investigated the relationship between cannabis or cigarette use (at age 16) and diagnosis of depression or anxiety 2 years later (115). The authors determined that after adjusting for potential confounds and cigarette use, the relationship between cannabis use and anxiety symptomology diminished to a non-significant result. Similarly, in another population-based cohort design spanning 30 years, cannabis use in adolescence predicted depressive symptomology and suicidality at age 50, but not anxiety (113). Two other prospective cohort designs also determined that cannabis use in adulthood was not associated with anxiety symptomology, however these studies did not obtain or consider information regarding adolescent cannabis use (127, 135).

Two cross-sectional designs comparing non-clinical cannabis users to non-users suggested a relationship between cannabis use and greater subclinical anxiety symptomology (54, 100). One study classified cannabis users into those demonstrating presence of CBD in hair and those who did not, in addition to high- or low- THC levels. Users with high-THC levels in their hair and users with no CBD reported the most depressive and anxious symptomology, suggesting negative long-term effects of high-THC on mood (54). Wright et al. (100) investigated whether adult, non-clinical cannabis users differ from non-users in self-reports of anxiety, depression, and behavioral approach to rewards. In line with their hypothesis, users reported elevated depressive symptoms, and female users reported elevated anxiety symptoms than non-users.

Seven experimental studies in which healthy non-users received varying doses of THC produced greater anxious symptomology in comparison to placebo (13–15, 41, 52, 53, 90). McDonald et al. (90) administered either 7.5 or 15 mg of THC to a sample of men and women with no history of a psychiatric disorder prior to a neurocognitive battery and found a dose-dependent effect of THC on anxiety, anger, fatigue, and confusion. A more recent study provided recreational cannabis users either placebo (0 mg), 29, 49, and 69 mg of THC and obtained a dose-dependent effect of THC on increasing levels of anxiety (52). Additionally, the researchers found that subjective effects persisted up to 8 h post-intoxication.

Finally, two studies administering THC to volunteers without a history of a psychiatric disorder found no effects of THC on anxious symptomology while an additional two studies suggested an anxiolytic effect of CBD among a sample of healthy participants (61, 122, 134, 136). Zuardi et al. (136) and Linares et al. (134) both found that 300 mg of CBD reduced adult participants' self-report of speech-induced anxiety in comparison to placebo, 150 or 600 mg of CBD.

Overall, the literature implicates a dose-dependent relationship between greater levels of cannabis use and elevated anxious symptomology. However, the evidence suggests acute, anxiolytic effects of CBD, a constituent of cannabis sativa.

### Psychosis

Thirteen longitudinal designs concluding a relationship between cannabis use and an increased risk for the development of a psychotic disorder were included in this review (124, 132, 138, 141–144, 147–152). One cohort study following Swedish male conscripts at ages 18–20 for 27 years obtained a dose-dependent relationship between cannabis use and a formal diagnosis of schizophrenia (143). However, the study was limited in that data regarding use of cannabis before conscription was unavailable. A more recent, nationally representative, birth cohort design (*N* = 6,534) also identified a dose-dependent, positive relationship between adolescent cannabis use and psychosis in adulthood (151). Cannabis use between the ages of 15–16 years was associated with a subsequent psychosis diagnosis by age 30, and this effect persisted after controlling for baseline prodromal symptoms, daily smoking, alcohol use, other substance use, and parental psychosis. A separate cohort design found that cannabis use at age 16 predicted psychotic symptoms at age 19 (152). However, psychotic symptoms at age 13 predicted cannabis use at, respectively, ages 16 and 19, providing support for a bidirectional causal association between the two variables. Only one cohort design did not obtain a significant relationship between cannabis use and an increased risk for psychosis after adjusting for relevant confounders, including tobacco use (153).

Two cross-sectional studies comparing cannabis users and non-users without a psychiatric disorder concluded that users report greater psychotic symptoms than non-users (54, 137). Morgan et al. (54) classified cannabis users via hair samples into those who use both THC and CBD, and those who only use THC. Cannabis users who only demonstrated use of THC reported significantly more psychotic symptomology than THC and CBD users and non-users, implicating a relationship between THC and psychotic symptomology. Similarly, a more recent cross-sectional design attained a dose-dependent relationship between cannabis use frequency and severity of psychotic symptoms including mania, paranoia, and presence of auditory hallucinations (137). The effects persisted even after adjusting for relevant confounds, such as sex, age, and other substance use (137).

One pilot, within-subjects, placebo-controlled, double-blind design obtained no effect of acute THC administration on psychotic, anxious, or depressive symptomology following a 5 day pre-treatment of THCV (61). An earlier study led by the same team of researchers also found that pre-administration of CBD reduced participants' self-reports of psychotic symptomology on the Positive and Negative Affect Syndrome Scale (PANSS) (140). However, relative to placebo, this difference did not reach significance, suggesting that CBD may not fully attenuate the psychotic symptoms produced by THC.

Overall, the literature presents strong evidence that acute THC intoxication increases reports of psychotic symptomology. Moreover, the literature suggests a dose-dependent relationship between levels of cannabis use and increased risk for the development of a psychotic disorder.

## Discussion

### Cognition

Concerning cognitive impairments, there is moderate evidence that chronic cannabis use and acute THC intoxication may negatively impact verbal, working, and episodic memory, executive functioning, and divided and sustained attention in users. However, cannabis does not appear to affect all cognitive domains, as impairments in visuospatial memory, processing speed, inhibitory control, and IQ were less consistent with mixed results.

Our review suggests a dose-dependent relationship between chronic cannabis use and verbal, episodic, and working memory impairments, but primarily among individuals who began use in adolescence. These findings coincide with preclinical animal models which have found that repeated exposure to THC during adolescence, but not adulthood, negatively impacts multiple cognitive domains, including memory, throughout the lifespan (156, 157). We also found modest evidence for improvements in cognition with cannabis abstinence, as some studies comparing non-users and past users demonstrated similar levels of performance among laboratory assessments (36, 65, 66). Given the brain's plasticity, restoration of neurocognitive abilities may be expected. A recent meta-analysis investigating residual cognitive impairments in cannabis users found no significant deficits among individuals who had abstained for at least 25 days (158). However, additional well-controlled prospective designs monitoring cognition from current use through cessation of use and over extended periods of abstinence are needed.

Among studies investigating the effects of acute THC administration, the evidence implicates greater impairment in cognition among recreational cannabis users and non-users in comparison to chronic cannabis users [e.g., (48, 61, 70, 79)]. In fact, a recent review evaluating the development of tolerance among cannabis users obtained comparable findings suggesting that cognition was most impaired upon acute THC intoxication (159), suggesting minimal tolerance.

Despite these findings, mixed evidence for numerous cognitive domains in this review arose, which may be due to variability in the control variables employed, cognitive tests utilized, operationalization of cognitive domains, participants' cannabis use histories, and cannabis exposure heterogeneity. Irrespective of these limitations, the effects obtained in this systematic review suggest that impairment of numerous cognitive domains can persist well-beyond the period of acute intoxication and consequently adversely impact everyday functioning in cannabis users.

### Motivation

Although the research concerning the effects of cannabis on motivation among non-clinical populations is limited, the evidence suggests a moderate negative relationship between cannabis use and motivation. These findings align with the “amotivational syndrome,” a term first coined by Smith [(160), p. 43] which purports that individuals who use cannabis are characterized by “a loss of desire to work or compete”, in addition to reduced emotional reactivity and interest in attaining goals (161).

While cannabis use may adversely impact motivation, the results need to be interpreted with caution due to certain methodological limitations. All of the included studies were cross-sectional, which significantly limits our understanding concerning the directionality between these two variables. Moreover, the conception and operationalization of motivation greatly differed across studies. While some of the included studies employed self-report measures such as the Apathy Evaluation Scale (AES) to measure apathy, other studies utilized performance-based tasks and operationalized motivation as perseverance in working for a monetary reward. Despite these methodological limitations, the evidence concerning cannabis use and reduced motivation aligns with studies suggesting that chronic cannabis users demonstrate reduced occupational and educational attainment compared to non-users [e.g., (107, 111, 112)]. Nevertheless, the field can significantly benefit from controlled, large-scale prospective designs evaluating motivation throughout the life trajectory while considering the impacts of the frequency of cannabis use, age of onset for use, and the influence of other substance use on motivational outcomes.

### Psychosocial Functioning

With a global trend toward legalization and decriminalization of cannabis for medical and/or recreational uses, the potential psychosocial harms accompanying cannabis use remain a major public health concern. Use of cannabis by adolescents is widespread and our findings suggest that chronic cannabis use throughout this developmental period is strongly associated with reduced educational and occupational attainment among this population. Nevertheless, the mechanisms linking cannabis and educational and economic risks are unclear. However, early age of onset, frequent or heavy use, and predictors of early use (e.g., childhood conduct or depressive symptoms), may undermine educational and occupational attainment. Consequently, delaying use onset and reducing the frequency of cannabis use patterns among adolescents may be beneficial in minimizing disruptions to educational goals and economic success.

While much of the evidence focuses on post-secondary or high school educational attainment, only one study included in the review followed a birth cohort until middle adulthood (107). The authors found that persistent cannabis use throughout adulthood corresponded with adverse mental health, substance use, and psychosocial outcomes, even after controlling for relevant confounding factors. Moreover, the authors denoted individual and childhood factors predicting persistent cannabis use in adulthood, including novelty-seeking, parental substance use, deviant peer affiliation, and conduct disorder diagnosis in adolescence. While additional, well-controlled studies are required to substantiate these findings, regardless of causality, our review indicates that individuals who utilize heavy amounts of cannabis for an extended period may experience adverse consequences to their social and economic well-being throughout the lifespan.

### Psychiatric Outcomes

#### Depression and Anxiety

The review obtained mixed evidence concerning the relationship between cannabis use and depressive symptomology. Numerous studies implicated a dose-dependent relationship between cannabis use beginning in adolescence and increases in depressive symptomology. However, this effect was inconsistent across studies, which may be attributed to methodological differences within the literature. For example, differences surrounding the follow-up period and assessments of cannabis use frequency and levels may have precluded other authors from distinguishing a relationship between cannabis use and depressive symptomology. Moreover, the reported association between cannabis use and depression may have been influenced by variation among the controlled variables across the studies reviewed. A modest proportion of the cohort studies obtaining a significant relationship between adolescent cannabis use and increased depressive symptomology in adulthood did not account for additional substance use, such as nicotine and alcohol [e.g., (117, 118)]. This is of significance because alcohol and tobacco use are prevalent among cannabis users, and these substances may independently elevate individual susceptibility to depression if used throughout adolescence (162–164).

Concerning the effects of cannabis use on anxious symptomology, while individuals frequently report cannabis as an effective agent to relieve anxiety, [e.g., (165)] the evidence suggests a moderate dose-dependent relationship between cannabis use and heightened anxious symptomology. One potential exception surrounding these findings is in the case of cannabidiol (CBD). Among two randomized, double-blind, placebo-controlled, experimental designs, relative to placebo, CBD reduced anxiety on a social stress test among a sample of non-clinical participants (134, 136). While these findings implicate an anxiolytic effect of CBD, future research is necessary to evaluate whether these effects persist long-term.

Despite these findings within the anxiety literature, there are some noteworthy methodological considerations. Similar to the findings surrounding cannabis and depression, controlled factors varied across studies. Several cohort designs did not obtain significant relationships between cannabis use and anxiety disorders after controlling for other substance use and childhood psychosocial variables [e.g., (117, 127, 135)], while studies that did not control for one or more of these variables did obtain significant findings [e.g., ((63, 129)]. Secondly, follow-up times of cohort designs significantly varied, ranging from 1 to 35 years, with attention directed toward adolescents and young adults. Consequently, the effects of cannabis use on anxious symptomology during middle and late adulthood are poorly understood.

Although the review suggests cannabis as heightening anxious symptomology and possibly depressive symptomology, the mechanism underlying this relationship has not been clearly established. A neurobiological explanation has been put forward suggesting that THC may perturb endocannabinoid system CB1 receptor signaling, which has been linked to psychopathology and dysregulation of emotional experiences (166). Animal models have also demonstrated that administering THC during adolescence elevates symptoms reflecting anhedonia and anxiety in adulthood and is paralleled by neurotransmitter changes, including a diminution in serotonin, which is a neurotransmitter linked to depression, and increases in norepinephrine, which is a neurotransmitter linked with anxiety (167, 168). Interestingly, a recent preliminary study of 28 days of cannabis abstinence in people with major depression and cannabis use disorder suggests clinically relevant improvements in depression, anxiety and motivation (169).

A second explanation for the relationship between cannabis and elevated anxious and depressive symptomology utilizes a psychosocial lens (170). Cannabis use is associated with numerous adverse psychosocial outcomes, including unemployment, increased affiliation with deviant peers, and poorer educational outcomes (106, 112), which are all factors that may increase risk of developing an anxious or depressive disorder.

#### Psychosis

We found a strong relationship between chronic cannabis use and an increased risk for psychosis. This relationship persisted independent of alcohol [e.g., (143, 153)] and tobacco [e.g., (124, 138, 152)] use. In comparison to non-users, cannabis users have an earlier age of onset of psychotic disorders (143, 147, 151). Moreover, the association between cannabis use and psychotic symptomology is elevated with heavier, more frequent, and earlier use (27, 132, 138, 143). These findings coincide with a large meta-analysis which analyzed over 20,000 subjects, and found that the onset of psychosis is 2.7 years earlier in cannabis users than in non-users (171).

Although the evidence supports a relationship between cannabis use and psychotic symptoms, these findings may be confounded by tobacco use, as a significant proportion of cannabis users also smoke cigarettes. Moreover, many longitudinal studies observing a relationship between cannabis use and increased psychotic symptomology or a diagnosis of a psychotic disorder, had not recorded tobacco use [e.g., (138, 144, 148)]. A recent meta-analysis found that daily tobacco use correlates with an increased risk of psychosis, in addition to an earlier onset of a psychotic disorder (172). However, other evidence suggests that acute nicotine or tobacco use does not exacerbate the positive and negative symptoms of psychosis in schizophrenia [e.g., (173, 174)], and that abstinence does not alter schizophrenia psychosis (175).

Despite the potential confounding role of tobacco use in longitudinal designs, direct evidence obtained within experimental studies demonstrate a clear temporal association between THC-intoxication and increased psychotic symptomology, including positive, negative, and cognitive symptoms [e.g., (24, 53, 146)]. Further, reports of psychosis within randomized, placebo-controlled, experimental studies of THC administration are commonly made, and among some individuals, psychosis persists beyond the acute intoxication phase (15, 176, 177). Therefore, the primary symptom clusters present in schizophrenia are also frequently present in varying degrees during THC-intoxication.

### Overall Strengths and Limitations

One of the major strengths of this review includes its behavioral focus: we addressed important questions concerning the clinical, cognitive, and psychosocial outcomes associated with cannabis use. Our findings provide evidence that there are numerous risks associated with cannabis use, which is likely of significant interest to public health officials, educators, policymakers, researchers, healthcare practitioners, and the general public. Additionally, extending our review to a broad array of outcomes allowed us to detect major gaps in the current literature. A final strength of this review is that we employed a methodologically rigorous and comprehensive approach in collecting our evidence by following PRISMA guidelines (32).

Although we performed a comprehensive, systematic review, there are important limitations to note. As previously discussed, many of the studies across the investigated domains did not control for important confounding factors such as alcohol, tobacco and other substance use, or familial and other psychosocial variables [e.g., (58, 63, 74, 129)]. Accordingly, the possibility that the negative impacts of cannabis use upon the outcomes explored are attributed to confounding factors cannot be dismissed. An additional limitation of the present systematic review is the heterogeneity of the included studies insofar as study methodology, outcome measures assessed, and duration of follow-up. Participants differed on various socio-demographic characteristics and cannabis use parameters, including age of onset, lifetime use, abstinence periods for former users, and frequency of use. Moreover, the potency of cannabis used, and relative concentrations of THC and CBD are also important to consider and were infrequently discussed in the included studies. These methodological differences have likely contributed to the mixed findings, and should be addressed in future research.

Despite these limitations, we did identify a trend where frequent or heavy cannabis use in adolescence was typically a significant risk factor for numerous adverse outcomes in adulthood, including worsened educational attainment, reduced IQ, and the development of an anxiety disorder, major depressive disorder and psychosis (See Table 2 for a summary of the quality of the evidence).

**Table 2:** The strength of evidence concerning cannabis and cannabinoids in behavioral outcomes among persons without a medical condition or history of a psychiatric disorder.

**Behavioral Outcome**	**Number of studies finding a negative impact of cannabis use**	**Number of studies finding no impact of cannabis use**	**Level of Evidence**
Verbal, Episodic, and Verbal Working Memory	24	13	**4** (24/37 = 64.9%)
Visuospatial Memory	1	6	**1** (1/6 = 16.7%)
Attention	12	8	**4** (12/20 = 60.0%)
Processing Speed	3	3	**3** (3/6 = 50.0%)
Executive Function	16	8	**4** (16/24 = 66.7%)
Impulsivity/Inhibitory Control	10	7	**3** (10/17 = 58.8%)
Intelligence (IQ)	3	4	**3** (3/7 = 42.9%)
Motivation	4	2	**4** (4/6 = 66.7%)
Psychosocial Functioning	7	1	**5** (7/8 = 87.5%)
Depression	16	11	**3** (16/27 = 59.3%)
Anxiety	14	9	**4** (14/23 = 60.9%)
Psychosis	25	2	**5** (25/27 = 92.6%)

*1 = 0–19% corresponds with strong evidence of no effects of cannabis use*.

*2 = 20–39% corresponds with moderate evidence of no effects of cannabis use*.

*3 = 40–59% corresponds with mixed evidence of neutral or negative effects of cannabis use*.

*4 = 60–79% corresponds with moderate evidence of negative effects of cannabis use*.

*5 = 80–100% corresponds with strong evidence of negative effects of cannabis use*.

Our findings suggest that the adolescent brain is especially vulnerable to the effects of cannabinoids (especially THC) in comparison to the adult brain. Prior research has demonstrated that exposure to endogenous cannabinoids modifies the endocannabinoid system, which is a major player in shaping neurodevelopmental processes, including modulating neuroplasticity and regulating synaptic connections (178–180). It is possible that cannabis use during adolescence disrupts these neurodevelopmental processes. Consequently, this may produce enduring changes in brain structure and function that underlie many of the adverse cognitive and clinical outcomes associated with adolescent cannabis use.

## Conclusion and Future Directions

The use of cannabis is extensive, ranging from occasional use to daily use and CUD. Although the desired effects sought by recreational cannabis users include relaxation, euphoria, and decreased anxiety, our review obtained evidence of adverse, acute and chronic sequelae of cannabis use, including impairments in cognition, increased risk for psychosis, depression, and anxiety, and poorer psychosocial functioning.

Although the evidence obtained in this systematic review implicates numerous adverse consequences of cannabis use beginning in adolescence, cannabis use among this age group is increasing (5, 181, 182). Moreover, with Canada's recent legalization of recreational cannabis and as more US states (now 10 states plus the District of Columbia) and nations consider legalizing medical or recreational cannabis use, the perceived risk of cannabis has also been trending downward cite (6, 183). Consequently, further investigation of the effects of these policies on usage patterns and related outcomes are a major public health concern.

Future research investigating the impact of cannabinoids during aging processes in middle and late adulthood is strongly needed, as little attention has been paid to this demographic. This is of concern because cannabis use is also increasing among this population (184), yet the effects of this substance in late adulthood are unknown. These studies will be crucial in depicting a more complete picture concerning the replicability and robustness of observed effects.

Finally, our work highlights a clear need for well-controlled longitudinal cohort and experimental vs. cross-sectional designs that utilize standardized measures of cognition, intelligence, motivation, psychiatric symptoms, and psychosocial functioning. Future studies should also be of adequate duration to assess cannabis and constituent effects, and have adequate follow-up periods to evaluate cannabis abstinence effects on these outcome measures. This would permit more accurate measurement of cannabis-related effect sizes on these outcomes. Such refinements in future methodologies would allow rigorous meta-analyses of cannabis effects on these various behavioral sequelae which may ultimately inform clinical and political decision-making.

## Data Availability Statement

The original contributions presented in the study are included in the article/Supplementary Material, further inquiries can be directed to the corresponding author/s.

## Author Contributions

MS and TG conceived and designed the presented idea. MS and RB were involved in collecting data and designing the tables. MS wrote the manuscript with support from TG. TG encouraged MS to investigate specific topics for the review and supervised the findings of this work. All authors contributed to the article and approved the submitted version.

## Conflict of Interest

The authors declare that the research was conducted in the absence of any commercial or financial relationships that could be construed as a potential conflict of interest.
