# Supplementary material for: The Behavioral Sequelae of Cannabis Use in Healthy People: A Systematic Review
Source: Front Psychiatry. 2021 Feb 16;12:630247. doi: 10.3389/fpsyt.2021.630247 (PMC7920961; doi:10.3389/fpsyt.2021.630247)
Supplement: Supplementary file 1 [file Table_1.DOCX]

*Table 1: Characteristics of included studies for review.*

| (Author, Year) | Participants (age, participant group, sample size) | Methods (Study design, follow-up time, Measurements of Outcome Variable) | NOS Rating | Outcome Variable(s) | Matched OR Controlled Variables | Level of Cannabis Use^1^ | Relevant Findings |
| --- | --- | --- | --- | --- | --- | --- | --- |
| (Ansell, Laws, Roche, & Sinha, 2015) | US adults (18-55); Current cannabis users (N=43) | Ecological momentary assessment (14-day); Barratt Impulsiveness Scale-Brief | N/A | Impulsivity | Age, gender, alcohol and tobacco consumption | Recreational | -Self-reported cannabis use was associated with increased impulsivity on the same and following day in comparison to days when cannabis was not used |
| (Arkell et al., 2019) | US adults (18-65); light cannabis users (N=14) | Randomized, double-blind, within-subjects crossover design; (125mg THC, 62.5 mg THC and 62.5 CBD, or placebo); Digit Symbol Substitution Task (DSST); Divided Attention Task (DAT); Paced Auditory Serial Addition Task (PASAT) | N/A | Processing speed and attention | Not reported | Recreational | - THC and THC/CBD conditions both produced significant impairments in attention and processing speed in comparison to placebo |
| (Arseneault et al., 2002) | New Zealand birth cohort; national representative sample (N=759) | Prospective, birth cohort longitudinal design (follow-up at ages 11, 15, 18, 26**)**; Structured Clinical Interview for DSM-IV | 8 | Symptoms of  schizophrenia and depression,  diagnoses of  schizophreniform disorder and depression | Socioeconomic status, sex, parental education level | N/A | -Cannabis use before or at age 15 predicted schizophrenia symptoms, or a diagnosis for schizophrenia and schizophreniform disorder at age 26.  -Cannabis use did not predict depressive outcomes at age 26 |
| (Assari, Mistry, Caldwell, & Zimmerman, 2018) | American black adolescents at baseline (13-16) national representative sample (N=681) | -Longitudinal cohort design (3-yr follow-up); Brief Symptom Inventory | 8 | Depression | Age, gender, family SES | N/A | -Baseline cannabis use predicted an increase in depressive symptomology for adolescent boys but not girls  -Baseline depressive symptoms did not predict subsequent cannabis use |
| (Atakan et al., 2013) | UK male adults, (20-42); naïve cannabis users (N=21) | double-blind, placebo-controlled within-subject design; 10mg THC or placebo; battery of clinical assessments, go/no-go task | N/A | Psychotic symptoms, inhibition | Cigarette, alcohol, cannabis, and other drug use | Controls | -Intoxication led to significantly greater reports of positive and negative schizophrenic symptomology in comparison to placebo  -Participants reporting greater psychotic symptoms during intoxication displayed significant impairments in inhibitory control |
| (Baggio et al., 2014) | Swiss male young adults (18-22); national representative sample (N=5084) | Longitudinal, cohort design (2-yr follow-up); Cannabis Use Disorder Identification Test (CUDIT); World Health Organization–Major Depressive Inventory, | 6 | Depressive symptoms | Not reported | N/A | -Cannabis use at Time 1 predicted depressive symptoms at Time 2 in a dose-dependent manner |
| (Bechtold, Hipwell, Lewis, Loeber, & Pardini, 2016) | US adolescent and pre-adolescent boys at baseline (5-12); national representative sample (N=1009) | Longitudinal cohort design (annual follow-up from ages 13 - 18); Substance  Use Questionnaire; Youth  Self Report | 7 | Psychosis | Other substance use | N/A | -For each year adolescent boys engaged in regular  cannabis use, their expected odds of subsequent subclinical  psychotic symptoms rose by 21% and their expected odds of  experiencing subsequent subclinical paranoia or hallucinations rose by 133% and 92% |
| (Becker et al., 2010) | German young adults (18-25); early-onset and late-onset cannabis users (N=43) | Counterbalanced, cross-sectional design; verbal n-back task | N/A | Verbal memory | Tobacco use, education | Chronic | -No behavior differences between groups emerged |
| (Becker et al., 2018) | US Adolescents (19-20); chronic daily cannabis users and non-cannabis controls (N=73) | Cohort, Longitudinal (2yr follow-up); Neurocognitive battery | 8 | Motor function, speeded attention, verbal fluency, verbal working memory, verbal learning, spatial memory, planning, motivated decision-making | IQ, age, sex, alcohol use | N/A | - At follow-up, cannabis users demonstrated impairment in working memory, planning, and verbal memory.  - Cannabis users did not demonstrate impairments in speeded attention, spatial memory, or decision-making |
| (Bhattacharyya et al., 2009) | US white, male, adults (18-55); naïve cannabis users, (N=15) | double-blind,  randomized, placebo-controlled, repeated-measures,  within-subject design; 10mg THC, 600mg cannabidiol, or placebo; verbal learning task, battery of clinical assessments | N/A | Verbal learning, anxiety, psychotic symptoms | Not reported | Controls | -THC intoxicated participants did not demonstrate impairments in verbal learning  -THC intoxicated participants reported greater levels of anxiety and psychotic symptoms than placebo or cannabidiol |
| (Blanco et al., 2016) | US adults at baseline (18-24); national representative sample (N=34663) | Longitudinal, cohort, prospective design (3yr follow-up); Alcohol Use Disorder and Associated Disabilities  Interview Schedule | 7 | Psychiatric disorders | Age, sex, divorce history, education, race, family environment, family health history, | N/A | -Cannabis use at baseline did not predict any mood or anxiety disorder at follow-up |
| (Blest-Hopley, O’Neill, Wilson, Giampietro, & Bhattacharyya, 2019) | UK adults (18-55); adolescent-onset cannabis users, non-users (N=42) | fMRI cross-sectional design; paired associate verbal learning task | 9 | Verbal learning and memory | Substance use, age, education | Chronic | -Cannabis users demonstrated learning impairments in comparison to non-users |
| (Bloomfield, Morgan, Kapur, Curran, & Howes, 2014) | UK adults (18-55); regular cannabis users and non-users (N=38) | PET cross-sectional, design; Apathy Evaluation Scale | 7 | Apathy | Current cannabis use, tobacco and ecstasy use | Recreational, Chronic, and Dependent | -All users reported significant levels of apathy  -There were no significant associations between apathy scores and current cannabis use and age of first cannabis use |
| (Boccio & Beaver, 2017) | US adolescents at baseline (13-22); National representative sample (N=6584) | Cohort, longitudinal design (5yr follow-up); Picture Vocabulary Test | 7 | IQ | Sex, age, SES, race | N/A | -Cannabis use in adolescence (13-22) was a risk factor for a 1-2-point decline in IQ by the 5-yr follow-up (18-26) |
| (Boden, Dhakal, Foulds, & Horwood, 2020) | New Zealand birth cohort; national representative sample (N=1065) | Longitudinal, birth cohort (follow-up annually until age 16, then ages 18, 21, 30, and 35); DSM-IV interview, social/family questionnaires, socioeconomic questionnaires | 9 | DSM-IV disorders, socioeconomic outcomes, social/family outcomes | childhood, family and individual characteristics | N/A | -Heavy and persistent cannabis use in adulthood was a risk factor for major depression, anxiety disorder, suicidal ideation, psychotic symptoms, welfare dependence, and lower SES |
| (Boggs et al., 2018) | US adults (18-55); previous cannabis users (N=23) | within-subject, double-blind, randomized, cross-over, and counterbalanced study; intravenous THC (placebo, 0.015 mg/kg, and 0.03 mg/kg); neurocognitive battery | N/A | Fine motor control, sustained attention, and motor timing | Alcohol use, caffeine use | Recreational, Chronic, and Dependent | Intoxicated participants did not demonstrate impairments in sustained attentions  -intoxicated participants demonstrated dose-dependent deficits in fine motor control and timing |
| (Bolla, Brown, Eldreth, Tate, & Cadet, 2002) | US Adults (10-35); light, medium, and heavy cannabis users (N=22) | Repeated-measures design; 28-day abstinence program; neurocognitive battery (tests at 0, 1, 7, and 28 days) | N/A | Memory, executive function, psychomotor speed, manual dexterity | -Age, sex/gender, IQ | Chronic and Dependent | -Heavy cannabis users demonstrated impairments in memory and executive function despite abstinence |
| (Bolla, Eldreth, Matochik, & Cadet, 2005) | US Adults (21-40); cannabis users and non-cannabis controls (N=22) | Cross-sectional design; Iowa Gambling Task | 8 | Decision-making | IQ scores, education, SES, race, alcohol use, and sex/gender | Chronic and Dependent | - Cannabis users demonstrated impairment in decision-making in comparison to controls |
| (Borgwardt et al., 2008) | Swiss male adults (20-42); naïve cannabis users (N=15) | double-blind, pseudo-randomized, placebo-controlled repeated measures within-subject design; 10 mg THC, 600 mg CBD, placebo; Go/No-Go task | N/A | Impulsivity | Not reported | Controls | - No behavior differences between conditions emerged |
| (Burggren et al., 2018) | US older adults (64+); former chronic cannabis users and non-users (N=40) | Case-control design; neurocognitive battery | 9 | Verbal memory, processing speed, executive functioning | education, cigarette usage, | Former chronic users and non-users | - No behavior differences between groups emerged |
| (Carey, Nestor, Jones, Garavan, & Hester, 2015) | Irish adults (18-34); chronic cannabis users and non-users (N=15) | Cross-sectional design; paired associate learning task | 9 | Verbal learning and memory | Education, alcohol use | Recreational, Chronic, and Dependent | -Cannabis users demonstrated significant impairments in both verbal learning and memory |
| (Casey & Cservenka, 2020) | US adolescents (18-22); cannabis users and non-users (N=65) | Cross-sectional design task; Iowa Gambling Task | 8 | Decision-making | Age, IQ | Chronic, Dependent | -Cannabis users demonstrated significant impairments in decision-making than non-users |
| (Caspi et al., 2005) | Dunedin birth cohort, national representative sample (N=803) | Birth cohort longitudinal design (21yr follow-up); Diagnostic Interview Schedule; COMT Genotyping | 9 | Psychosis, psychiatric symptoms, COMT gene | IQ, Conduct Disorder | N/A | - Carriers of the COMT valine158 allele were most likely to exhibit psychotic symptoms and  to develop schizophreniform disorder at follow-up if they used cannabis during adolescence in comparison to individuals without this allele |
| (Castellanos-Ryan et al., 2017) | Canadian male boys at baseline (4-6); community sample (N=294) | Prospective longitudinal design (annual follow-up from ages 10 -17, and again at 20); neurocognitive battery | 9 | IQ, working memory, short-term memory, executive functioning, reward processing | -childhood adversity, academic achievement from 13 to 15 years verbal IQ at 13 years. Tobacco and other substance use, externalizing problems | N/A | -Early cannabis use was a significant risk factor for impairment in verbal IQ and executive function  -Early cannabis use was a significant risk factor for lower rates of high-school graduation |
| (Childs, Lutz, & de Wit, 2017) | US adults (18-55); naïve cannabis users (N=42) | Double-blind, randomized, and counterbalanced experimental design; placebo, 7.5-, 12.5 mg THC; Trier Social Stress Test and Primary Appraisal Secondary Appraisal rating scale | N/A | Psychosocial stress, mood, | Age, Trait anxiety, stress reactivity | Recreational and Controls | -Low-dose THC reduced subjective distress after stressful situations.  High dose THC increased negative mood overall |
| (Cousijn et al., 2014) | Denmark Adults (18-25); Heavy cannabis users and non-cannabis controls (N=73) | Quasi-Experimental; N‐back working‐memory task | 6 | Working memory | age, sex/gender, education, estimated intelligence | Chronic and dependent | -No behavior differences between groups emerged |
| (Curran, Brignell, Fletcher, Middleton, & Henry, 2002) | UK male young adults (18-30); naïve cannabis users (N=15) | Balanced, double-blind, cross-over design; 7.5mg and 15mg of THC, testing at pre- and 1, 2, 4, 6, 8, 24 and 48 h post-drug; Neurocognitive Battery; | N/A | Verbal learning, sustained attention, working memory, reaction time, verbal fluency | Not reported | Recreational | - THC was associated with impairments in explicit memory and learning impairments in a dose-dependent manner |
| (D’Souza et al., 2005) | US adults (18-55); naïve cannabis users (N=22) | 3-day, double-blind, randomized, placebo, and counterbalanced experimental design; 2.5, and 5 mg THC; Positive and Negative Symptom Scale, Clinician Administered Dissociative Symptoms Scale, Hopkins Verbal Learning Test, | N/A | Positive psychotic symptoms, negative psychotic symptoms, mood states, memory | Education, age | Recreational and Chronic | - THC increased anxious and positive and negative symptoms    -THC did not lead to impairments in verbal memory but did lead to impairments in working memory |
| (Danielsson, Lundin, Agardh, Allebeck, & Forsell, 2016) | Swedish adults (20-64); national representative sample (N=8598) | Longitudinal, cohort, prospective design (3yr follow-up); battery of clinical assessments | 6 | Anxiety Disorder, MDD | Sex, age, alcohol and illicit drug use, education, family tension, place of upbringing | N/A | - Cannabis use was not a risk factor for any anxiety disorder or major depressive disorder |
| (Degenhardt et al., 2013) | Australian adolescents at baseline (14-17); national representative sample (N=1943) | Longitudinal, cohort, prospective design (15yr follow-up); Revised Clinical Interview  Schedule (CIS-R) | 9 | Psychiatric disorders | Sex, parent’s education, other substance use, age, nationality, smoking status, tobacco use, education, marriage status, employment, welfare | N/A | -Adolescent cannabis use was not a risk factor for depression  -Daily cannabis use in adolescence was a risk factor for general anxiety disorder |
| (Despina et al., 2020) | Canadian adolescents at baseline (15); nationally representative sample (N=1606) | -prospective longitudinal design (annual follow-up for 5 years); Mental Health and Social Inadaptation Assessment | 9 | Depression, suicide ideation | Alcohol, tobacco, and other drug use, | N/A | -Cannabis use at baseline was not a risk factor for depression or suicide ideation  -Depression at age 15 was a significant risk factor for subsequent weekly cannabis use |
| (Desrosiers, Ramaekers, Chauchard, Gorelick, & Huestis, 2015) | US adults (18-45); regular and occasional cannabis users (N=25) | Two-way mixed quasi-experimental design; 54mg THC; divided attention task, n-back task, balloon analog risk task | N/A | Divided attention, spatial working memory, impulsivity, psychomotor function | Not reported | Chronic and recreational | -Occasional cannabis users demonstrated impairment in psychomotor function and divided attention  -Occasional and regular users did not demonstrate impairments in working memory or risk-taking during intoxication |
| (Dougherty et al., 2013) | US adolescents (14-17); cannabis users and non-users (N=93) | Counter-balanced Quasi-experimental design; Barratt Impulsiveness Scale; Marijuana Craving Questionnaire; Neurocognitive battery | 8 | Attention. Memory, Decision-Making, Impulse Control, | Age, gender, ethnicity | Chronic and dependent | -Cannabis users reported significantly greater impulsivity than non-users  -Cannabis users demonstrated significant impairments in memory in comparison to non-users  -Cannabis users did not demonstrate impairments in decision-making or attention |
| (Duperrouzel et al., 2018) | US adolescents at baseline (14-17); national representative sample (N=250) | Longitudinal design; bi-annual assessments (1yr); Depression, Anxiety, Stress Scale | 6 | Anxiety | Current depressive symptomology, alcohol, and nicotine use | N/A | - Adolescent cannabis use was a significant risk factor for anxiety |
| (Englund et al., 2013) | UK adults (21-50); occasional cannabis users (N=48) | mixed within- and between-subjects double-blind, placebo, cross-over design; 600 mg CBD or placebo preceding 1.5 mg THC; State Social Paranoia Scale, Positive and Negative Syndrome Scale, University of Wales Mood Adjective Checklist, neurocognitive battery | N/A | Psychotic symptoms, affect, verbal learning and memory, processing speed, working memory, planning | Age, sex, BMI, other drug use | Controls | Post-THC, users in the CBD group reported significantly fewer positive psychotic symptoms and paranoia than the placebo pre-treated group  -Post-THC, users in the CBD group demonstrated less episodic memory impairments than the placebo pre-treated group |
| (Englund et al., 2016) | UK male adults, (21-34); naïve cannabis users (N=10) | randomized, double-blind, placebo-controlled, crossover design; 5-day 10mg THCV or placebo administration followed by 1mg THC IV administration, cognitive battery, battery of clinical assessments | N/A | Psychotic, anxious, and depressive symptoms, verbal learning and memory, processing speed, | Age, sex, BMI, other drug use | Controls | -Intoxicated participants demonstrated memory impairments  -Intoxicated participants did not report psychotic, anxious, or depressive symptoms |
| (Epstein et al., 2015) | US adolescents (10 at baseline); national representative sample (N=808) | Longitudinal cohort design (annual follow-up for 6 years, followed by assessments at ages 18, 21, 24, 27, 30, and 33); DSM-IV Interview Schedule; battery of clinical assessments | 6 | Substance Abuse and dependence, mental health disorder, problem behaviour, socioeconomic outcomes, positive functioning | Gender, ethnicity, childhood SES | N/A | -Cannabis use in adolescence was a significant risk factor for adverse mental health, educational, and economic outcomes |
| (Feingold et al., 2015) | US adults at baseline (18-65); national representative sample (N=43,093) | Cohort, prospective, longitudinal design (3-year follow-up); battery of clinical assessments | 8 | MDD and Bipolar Disorder | sex, age, educational level, household income, marital status, urbanity and region, substance use disorder, additional psychiatric disorder | N/A | - Cannabis use at baseline was not a significant risk factor for depression after adjusting for covariates  -Weekly and daily cannabis use was a significant risk factor for bipolar disorder after adjusting for covariates |
| (Feingold et al., 2016) | US adults at baseline (18-65); national representative sample (N=43,093) | Cohort, prospective, longitudinal design (3-year follow-up); battery of clinical assessments | 8 | Anxiety Disorders | sex, age, educational level, household income, marital status, urbanity and region, substance use disorder, additional psychiatric disorder | N/A | - Cannabis use was not a risk factor for any anxiety disorder |
| (Ferdinand et al., 2005) | Dutch children and adolescents at baseline (4-16) national representative sample (N=1580) | Birth cohort, prospective, longitudinal design (annual follow-up from ages 1-16, followed by assessments at ages 18, 21, and 25; Composite International Diagnostic Interview | 8 | Psychotic symptoms | Age, sex, | N/A | - Cannabis use was a significant risk factor for development of psychotic symptoms at follow-up  - Psychotic symptoms in individuals who never used cannabis was a significant risk factor for future cannabis use |
| (Fergusson, Horwood, & Ridder, 2005) | NZ birth cohort; national representative sample (N=1055) | Birth cohort, longitudinal design (25yr follow-up); battery of clinical assessments, | 8 | Psychotic symptoms | Socioeconomic status, family functioning, child abuse, gender, neuroticism, novelty seeking, self-esteem, child IQ, | N/A | -Daily cannabis use was a significant risk factor for development of psychotic symptoms at 21 and 25  - |
| (Fontes et al., 2011) | Brazilian adults (18-55); early-onset and late-onset cannabis users, non-users (N=148) | Counter-balanced, cross-sectional design; Wisconsin Card Sorting Test, Frontal Assessment Battery, Wechsler Adult Intelligence  Scale – Revised | 8 | Planning, conceptualisation, motor programming, divided attention, inhibitory control, visuospatial problem-solving, vocabulary | Age, education, IQ | Dependent | -Early-onset cannabis users demonstrated poorer cognitive  Performance, sustained attention, impulse control  and executive functioning. than controls and late-onset users |
| (Fridberg, Skosnik, Hetrick, & O’Donnell, 2013) | US Young adults (18-25); cannabis users and non-users (N=52) | Cross-sectional design; XO continuous performance task | 8 | Sustained and selective attention | Age | Chronic and Dependent | -No behavior differences between groups emerged |
| (Gage et al., 2014) | UK birth cohort, national representative sample (N=1756) | Prospective, population-based birth cohort design (T1 data at age 16, follow-up at age 18);  Psychosis-like-symptoms semi-structured interview | 6 | Psychotic experiences | Family medical history, maternal education, sex, urban living, age 8 IQ, bullying, peer problems, depressive and borderline symptomology, substance use, victimisation, personality | N/A | -Cannabis use was associated with a greater amount of psychotic experiences at age 16, but the relationship was attenuated after controlling for cigarette smoking frequency |
| (Gage et al., 2015) | UK birth cohort, national representative sample (N=1756) | Prospective, population-based birth cohort design (T1 data at age 16, follow-up at age 18); Clinical Interview Schedule-Revised | 6 | Anxiety Disorder, MDD | Family medical history, maternal education, sex, urban living, age 8 IQ, bullying, peer problems, depressive and borderline symptomology, substance use, victimisation, personality | N/A | - Cannabis use was a significant  risk factor for depression after adjustment for confounders  -Cannabis use was not a significant risk factor for anxiety after adjustment for confounders |
| (Gonzalez et al., 2012) | US adolescents and young Adults (17-24); cannabis users and non-users (N=130) | Cross-sectional design; Neurocognitive Battery | 8 | Impulsivity and Verbal Learning | Age | Chronic and dependent | - No impulsivity differences between groups emerged  - Cannabis users demonstrated impairment in verbal memory |
| (Griffith-Lendering et al., 2013) | Dutch adolescents at baseline (10-14); national representative sample (N=2120) | Prospective longitudinal design (Two 3-yr follow-ups); Youth Self-Report | 8 | Psychosis | Tobacco use, SES, parent medical history | N/A | -Cannabis use at age 16 predicted psychosis vulnerability at age 19  -Psychosis vulnerability at age 13 predicted cannabis use at ages 16 and 19 |
| (MFH Griffith-Lendering, Huijbregts, Mooijaart, Vollebergh, & Swaab, 2011) | Pre-adolescents at baseline (10-14) national representative sample (N=2230) | Prospective longitudinal design (3-yr follow-ups); Youth Self-Report | 8 | Internalizing and Externalizing behaviors | SES, parental psychopathology, other substance use | N/A | -Cannabis use was not a risk factor for anxiety or depression by age 16 |
| (Gruber, Dahlgren, Sagar, Gönenc, & Killgore, 2012) | US adults (18-55); adolescent-onset and adult-onset cannabis users, non-users (N=39) | fMRI cross-sectional design; Multi-Source Interference Task | 8 | Impulsivity | IQ, Age | Chronic and Dependent | - No behavior differences between groups emerged |
| (Gruber, Dahlgren, Sagar, Gönenç, & Lukas, 2014) | US adults (18-55); Early-onset cannabis users, late-onset cannabis users, and non-users (N=43) | fMRI cross-sectional design; Barratt Impulsiveness Scale | 8 | Impulsivity | age, IQ, days of alcohol use, socioeconomic status, nicotine use, | Chronic and Dependent | -Cannabis users reported significantly higher impulsivity scores than non-users |
| (Grunberg, Cordova, Bidwell, & Ito, 2015) | US young adults at baseline (18-21); frequent, infrequent, and non-cannabis users (N=338) | Longitudinal cohort design (3-yr follow-up); Temperament and Character Inventory; Adult Self-report | 6 | Anxiety, Depression | N/A | N/A | -Cannabis use was not a significant risk factor for increased anxiety or depression at follow-up |
| (Harder, Morral, & Arkes, 2006) | US adults at T1 of study (29-37) national representative sample (N=8759) | Cohort, longitudinal design (1-year follow-up from T1); Center for Epidemiologic Studies—Depression (CES‐D) questionnaire | 7 | Depression | race, gender, general health limitations, region of residence, urban versus rural residence, past‐year residence, criminal activity, past‐year marijuana use, family history, other drug use, education | N/A | -Overall cannabis use at baseline was not a significant risk factor for depression after adjusting for covariates  -Heavy cannabis use at baseline was a significant risk factor for depression after adjusting for covariates |
| (Hatchard, Fried, Hogan, Cameron, & Smith, 2014) | Canadian young adults (19-21); cannabis-users and non-users (N=24) | Cross-sectional; Counting Stroop Task | 7 | Executive Function | Nicotine use, SES | Recreational and chronic | -No behavior differences between groups emerged |
| (Hengartner, Angst, Ajdacic-Gross, & Rössler, 2020) | Swiss young adults at baseline (19-21); national representative sample (N=591) | Retrospective, longitudinal design (annual follow-up until 35/35, followed by assessments at 40/41 and 49/50); Structured Psychopathological Interview, Rating of the Social Consequences of Psychological Disturbances for Epidemiology | 9 | Depression, Anxiety, Suicidality, | SES, childhood substance abuse, family climate, education level, social support | N/A | -Early and frequent adolescent cannabis use was a significant risk for depression and suicidality at follow-up  -Cannabis use was not a significant risk factor for anxiety symptomology at follow-up |
| (Homel, Thompson, & Leadbeater, 2014) | Canadian adolescents at baseline (12-18); national representative sample (N=662) | Longitudinal, cohort, design (Five 2-year follow-ups); cannabis use, postsecondary enrolment interview, | 8 | Postsecondary education achievement | sex, maternal education, family structure, high school grades, conduct problems | N/A | - Regular cannabis users had the lowest high school grades, had more conduct problems and were least likely to enroll in post-secondary education in comparison to occasional- and non-users |
| (Hunault et al., 2014) | Dutch, male, adults (18-45); cannabis users (N=24) | - randomized, double-blind, placebo-controlled, crossover design; 29.3, 49.1 and 69.4 mg THC; Visual analogue scales | N/A | Memory,  concentration, sedation and anxiety | N/A | Recreational and Chronic | -THC-users reported significantly increased feelings of anxiety up to 8hr post-smoking  -THC users reported significant impairment in memory, concentration, and alertness |
| (Hayatbakhsh et al., 2007) | Australian birth cohort; national representative sample (N=3239) | Birth cohort, longitudinal design; (follow-up at 6 months, 5, 14, and 21); Young Adult Self-Report, Youth Self-Report | 8 | Anxiety disorder, MDD | Gender, maternal age and education, maternal marital status, family income, family health history, adolescent smoking status and alcohol consumption | N/A | -Cannabis use before 15 years was a significant risk factor for anxiety and depression |
| (Infante et al., 2020) | US adolescents at baseline (12-15); National representative sample (N=175) | Cohort, longitudinal design (14yr follow-up); Wechsler Intelligence Scale for Children; Customary Drinking and Drug Use Record; Hollingshead Index of Social Position | 9 | Visuospatial functioning, Inhibitory control, verbal memory, processing speed, | Age, sex, substance use | N/A | -Greater cumulative cannabis use in adolescence was a significant risk factor for impairments in inhibitory control and visuospatial functioning  -Cannabis use was not a significant risk factor for verbal memory or processing speed |
| (Jackson et al., 2016) | US pre-adolescent twins and triplets at baseline (9-11); national representative sample (N=2769) | Cohort, longitudinal design (8-yr follow-up); neurocognitive battery | 7 | IQ, crystallized intelligence, fluid intelligence | Age, sex, race, zygosity, and socioeconomic status | N/A | -Cannabis users had lower test scores relative to nonusers  and demonstrated a significant decline in crystallized intelligence by follow-up  -No dose-dependent relationships between cannabis use and IQ change was found  -Cannabis using twins did not differ significantly in their IQ decline relative to their abstinent siblings |
| (Jacobsen, Mencl, Westerveld, & Pugh, 2004) | US adolescents (14-19); cannabis users, tobacco users, non-users (N=21) | Cross-sectional fMRI design; Conner’s Continuous Performance Test, Word recognition task | 8 | Sustained Attention, Selective Attention, Divided Attention | Depression and anxious symptomology, inattention, stressful life events | Recreational and chronic | -Cannabis users demonstrated impairments in attention in comparison with tobacco users and non-users |
| (Jager, Block, Luijten, & Ramsey, 2010) | Dutch and US male adolescents (13-19); abstinent but frequent cannabis–using boys and non-users (N=45) | Cross-sectional fMRI design; Sternberg's item-recognition paradigm, associative memory task | 7 | Associative memory, working memory | age, estimated IQ, country (US versus NL); diagnosis of conduct disorder, cannabis use (frequency, abstinence); other substance use | Chronic and Dependent | -No behavior differences between groups emerged |
| (Jager, Block, Luijten, & Ramsey, 2013) | Dutch and US male adolescents (13-19); cannabis users and non-users (N=47) | Cross-sectional fMRI design; monetary incentive delay (MID) task | 7 | reward processing | age, estimated IQ, country (US versus NL); diagnosis of conduct disorder, cannabis use (frequency, abstinence); other substance use | Chronic and Dependent | No behaviour differences between groups emerged |
| (Jager, Kahn, Van Den Brink, Van Ree, & Ramsey, 2006) | Denmark Adults (18-55), current cannabis users and non-users (N=20) | Cross-sectional fMRI design; Sternberg item recognition task and visuo-auditory attention task | 9 | Working memory and selective attention | Age, gender/sex, IQ | Recreational and Chronic | -No behavior differences between groups emerged |
| (Kanayama, Rogowska, Pope, Gruber, & Yurgelun-Todd, 2004) | US Adults (30-55); heavy cannabis users and non-users (N=22) | Cross-sectional fMRI design; Spatial Working memory task | 8 | Spatial working memory | Race, education, gender/sex | Chronic and Dependent | - No behavior differences between groups emerged |
| (Kaufmann et al., 2010) | Austrian female adults (19-29); non-cannabis users (N=16) | Randomized, double-blind, active counterbalanced,  cross-over design; 20mg THC or 5mg diazepam; visual analogues scales, brief psychiatric rating scale | N/A | Psychotic symptoms, subjective high feelings | Not reported | Controls | -THC intoxication led to significantly greater reports of psychotic symptoms |
| (Kloft et al., 2020) | Dutch and Australian adults (18-65); occasional cannabis users (N=64) | Randomized, double-blind, placebo-controlled design; 300 μg of THC per kg of body weight; associative word lists and two misinformation  tasks using virtual reality | N/A | False memory under drug influence and 7-days later | Not reported | Recreational | -Intoxicated participants demonstrated higher false recognition at immediate and delay test than controls  - Intoxicated participants demonstrated higher false-memory creation at acute testing than controls |
| (Kuepper et al., 2011) | German adolescents and young adults at baseline (14-24); national representative sample (N=1923) | Prospective longitudinal design (10yr follow-up); Munich composite international diagnostic interview | 7 | Psychotic symptoms | Sex, SES, substance use, urban/rural environment, other psychiatric diagnosis | N/A | -Cannabis use at baseline was a significant risk factor for developing psychotic symptoms at follow-up |
| (Lane, Cherek, Pietras, & Steinberg, 2005) | US adolescent boys (14-18); cannabis users and non-users (N=34) | Cross-sectional design; progressive-ratio and fixed time motivation task | 7 | Motivation | Cognitive aptitude, gender, conduct disorder | Dependent | Cannabis users demonstrated significant impairment in motivation in comparison to non-users |
| (Linares et al., 2019) | Brazilian male adults (19-45); non-users (N=57) | Double-blind, randomized, placebo, and counterbalanced experimental design; placebo, 150 mg, 300 mg, or 600 mg CBD; stimulated public speaking task | N/A | Anxiety | Age, education, SES | Controls | -Intoxicated participants at 300mg of CBD demonstrated significantly less anxiety than intoxicated participants at CBD 150 mg CBD, 600 mg CBD, or placebo |
| (Lisdahl & Price, 2012) | US adolescents and young adults (18-26); cannabis users and non-users (N=59) | Cross-sectional design; Beck Depression Inventory-II; Timeline Follow-Back; Wide Range Achievement Test-4th edition; Neurocognitive Battery | 9 | Verbal memory, Psychomotor speed, sustained attention, verbal fluency, cognitive inhibition, design fluency | reading ability, gender, subclinical depressive symptoms, body mass index, substance use | Recreational, Chronic, and Dependent | - Cannabis use was associated with poorer psychomotor speed, sustained attention and cognitive inhibition in a dose-dependent manner in young adults  -Male cannabis users demonstrated greater cognitive impairments than female cannabis users  -Cannabis users did not demonstrate impairments in verbal memory |
| (Lovell et al., 2018) | Australian adults (18-65); abstinent cannabis users, long-term cannabis users, long-term tobacco users (N=35) | Quasi-experimental; Neurocognitive battery | N/A | Learning and memory, impulsivity, processing speed, sustained attention, | Age, education, lifetime tobacco consumption | Chronic and Dependent | -Cannabis users demonstrated significant impairment in learning and memory, processing speed, and response times in comparison to tobacco users and non-users  -Cannabis users demonstrated significant impairments in impulsivity in comparison to non-users |
| (Manrique-Garcia, Zammit, Dalman, Hemmingsson, & Allebeck, 2012) | Swedish, male, young adults at baseline (18-20); national representative sample (N=45087) | Cohort, longitudinal design (35-year follow-up); Battery of clinical assessments | 7 | Unipolar depression, Bipolar disorder, affective psychosis, schizoaffective disorder | IQ, deviant behaviors in childhood, social adjustment, alcohol use, nicotine use, SES, other drug use | N/A | -Overall, cannabis use was not a significant risk factor for future depression or completed suicide  -Cannabis use was a significant risk factor schizoaffective disorder |
| (Martin-Santos et al., 2012) | UK male adults (18-42); non-users (N=16) | Randomised, double-blind, cross-over, placebo-controlled design; 10mg THC, 600mg CBD, or placebo; battery of clinical assessments at 1, 2, and 3 hours post drug administration | N/A | Anxiety, dysphoria, positive psychotic symptoms, and physical and mental sedation | Not reported | Controls | - THC intoxication led to significantly greater reports of anxiety, dysphoria, positive psychotic symptoms, and physical and mental sedation in comparison to placebo and CBD |
| (Martin-Soelch et al., 2009) | Swiss adults (18-55); non-smokers, smokers, and cannabis users (N=53) | Cross-sectional, counterbalanced design; Spatial delayed response task; Mood ratings | 6 | Behavioral and affective response to reward | Age, education, Depressive symptomology | Chronic and Dependent | -Cannabis users rated their mood significantly lower than smokers and non-smokers during the task’s reward condition |
| (Matheson et al., 2020) | Canadian young adults (19-25); community sample (N=91) | Double-blind, placebo-controlled, parallel-groups design: High THC, low THC, or placebo cannabis: Profile of Mood States and neurocognitive battery before and 1, 24, and 48 (h) after intoxication | N/A | Mood, memory, processing speed, attention, and motor skills | Age, cannabis use frequency, IQ | Recreational and Chronic | -High-THC users reported increases in friendliness and elation, but an impairment in verbal memory  -Low and high-THC users demonstrated greater mood and improvement in processing speed |
| (McDonald, Schleifer, Richards, & de Wit, 2003) | US adults (18-45); naïve cannabis users (N=37) | Randomized, double-blind, placebo-controlled, within-subject design; placebo, 7.5, or 15 mg THC; Stop task, Go/no-go task, Delay discounting task; Profile of Mood States; The Addiction Research Center Inventory; Drug Effects Questionnaire | N/A | Impulsivity, mood, subjective states | Sex | Recreational and Chronic | - THC significantly increased subject ratings of, dysphoria, euphoria, anxiety, fatigue, anger, and confusion in a dose-dependent manner  -THC significantly impaired performance on 1 out of 4 tasks assessing Inhibitory control |
| (Medina et al., 2007) | US adolescents (16-18); 1-month abstinent cannabis users and non-users (N=65) | Cross-sectional design; Neurocognitive battery; | 9 | Psychomotor speed, Complex attention, Verbal Memory, Visuo-spatial memory, Verbal accuracy, Planning, Problem Solving | Depressive symptoms, substance use | Recreational, Chronic, and Dependent | -Abstinent cannabis users demonstrated impairments in complex attention, psychomotor speed, planning, sequencing ability, episodic memory, and verbal memory in comparison to nonusers  -Cannabis users did not demonstrate impairments in one domain of executive functioning, which was problem solving |
| (Meier et al., 2012) | Dunedin birth cohort; national representative sample (N=1748) | Prospective, birth-cohort longitudinal design (follow-up at ages 5, 7, 9, 11, 13, 15, 18, 21, 26, 32, and 38); Neurocognitive battery | 9 | IQ | Substance use, schizophrenia, past 24-h cannabis use and past-week cannabis use | N/A | -Persistent cannabis use was associated with neuropsychological and IQ decline  - Cannabis cessation did not fully restore neurocognitive functioning in adolescent-onset cannabis users |
| (Meier et al., 2015) | US adolescents at baseline (14-15); upper middle-income community (N=254) | Longitudinal cohort (annual 3-yr follow-up); Substance use frequency scale | 6 | Education attainment | Baseline GPA, delinquency, depression and anxiety symptoms. | N/A | -Persistent cannabis use across the four years of high school was a significant risk factor for lower grade-point average (GPA); lower Scholastic Aptitude Test (SAT) score, and greater externalizing symptoms  - |
| (Meier et al., 2018) | UK birth cohort; national representative sample of twins (N=2332) | Birth cohort longitudinal design (follow-up at ages 5, 12, and 18); Wechsler Preschool and Primary Scale of Intelligence‐Revised; Cambridge Neuropsychological Test Automated Battery | 8 | IQ | IQ at age 5, sex, co-twin variables, zygosity | N/A | -Cannabis use was not associated with IQ decline  -Twins who used cannabis more frequently than their co-twin demonstrated impairments in one test of working memory |
| (Meier et al., 2020) | US adolescent boys (15-16 at baseline) Community sample (N=856) | Cohort longitudinal design (annual follow-ups for 10 years); Substance Use Questionnaire, Youth Self  Report | 6 | Anxiety, depression | Substance use, subclinical psychotic symptoms, externalizing problems | N/A | -Weekly cannabis use was a significant risk factor for increases in depression and anxiety symptoms at follow-up |
| (Mercuri et al., 2018) | Australian Adults, (18-34); cannabis users and non-users (N=114) | Cross-sectional design; Autobiographical Interview | 6 | Episodic foresight and episodic memory | Intelligence and sex | Recreational and Chronic | - Regular cannabis users demonstrated greater impairment in episodic memory and episodic foresight than recreational cannabis users and non-users |
| (Miettunen et al., 2008) | Finnish birth cohort; National representative sample (N=6330) | Prospective, birth-cohort, longitudinal design (16yr follow-up); PROD-screen | 7 | Prodromal psychosis symptoms | Gender, childhood emotional and behavioural symptoms, SES, substance use, parental substance use disorder | N/A | Cannabis use before the age of 15 was a significant risk factor for reporting prodromal psychosis symptoms at follow-up |
| (Mokrysz, Landy, et al., 2016) | UK birth cohort; national representative sample (N=4621) | Longitudinal birth cohort (16yr follow-up); Wechsler Intelligence  Scale for Children; General Certificate of Secondary Education | 8 | IQ, education attainment | Maternal and early-life factors, childhood behavioural factors, childhood mental health, another adolescent drug use | N/A | -Frequent cannabis use was not a significant risk factor for lowered IQ or educational performance in comparison to occasional cannabis use or abstinence |
| (Mokrysz, Freeman, Korkki, Griffiths, & Curran, 2016) | UK male adolescents (16-17) and adults (24-28); cannabis users | Mixed within- and between-subjects, double-blind, cross-over design; 12% THC or placebo; Neurocognitive battery | N/A | spatial working memory, episodic memory, response inhibition | Premorbid intelligence, anxious and depressive symptom, body weight, age, | Chronic | -Adults demonstrated greater impairment in spatial working memory and episodic memory than adolescents  -Adolescents demonstrated greater impairments in impulsivity than adults |
| (Mokrysz et al., 2020) | UK adolescents and adults (16-70); cannabis users (N=57) | Study 1: within-subjects, double-blind, cross-over, placebo design; THC 12.0% CBD <0.1%; THC 6% CBD 7.5%  and placebo; Psychotomimetic States Inventory; White noise task  Study 2: mixed within- and between-subjects, double-blind, placebo, cross-over design; 12.0% CBD <0.1% and placebo; same measurements as Study 1 | N/A | Psychotic symptoms, speech illusions, perceptual distortion | Age | Recreational and Chronic | -In Study 1 and 2: Intoxicated participants reported increased psychotic symptoms relative to placebo  -In Study 1: CBD administration did not mitigate the impact of cannabis induced psychotic symptoms  -In Study 2: Intoxicated participants reported significantly greater speech illusions relative to placebo |
| (Morgan et al., 2012) | UK adolescents and young adults (16-23); daily and occasional cannabis users (N=120) | Cross-sectional, counter-balanced, design; Hair analysis; Brief Psychiatric Rating Scale, Schizotypal Personality Questionnaire; Prose recall, Source memory; Beck Depression Inventory; Spielberger State-Trait Anxiety Inventory | N/A | Psychotic symptoms, verbal memory, depression, anxiety | Baseline intelligence | Chronic | -Cannabis users with CBD levels in their hair reported less psychotic symptomology than non-CBD users  -Cannabis users with high THC levels in their hair reported increased depression and anxiety than users with low THC levels  -Daily users with high THC levels demonstrated significant impairments in verbal memory |
| (Morgan et al., 2010) | UK adolescents and young adults (16-23); cannabis users (N=134) | Repeated measures (7-day) design; Six days drug-free, one day intoxicated; Prose recall task; Psychotomimetic States Inventory | N/A | Verbal memory and psychotic symptoms | Education and age | Recreational and Chronic | - Cannabis users who smoked high levels of cannabidiol demonstrated no verbal memory impairment while users who smoked low levels of cannabidiol demonstrated verbal memory impairments  -Overall, cannabis users demonstrated increased psychotic symptoms under intoxication |
| (Morrison & Stone, 2011) | UK male adults (18-55); Naïve cannabis users (N=22) | Randomized, double-blind, active counterbalanced,  cross-over design; 2.5mg THC or placebo; Community Assessment of Psychic Experiences, Scale for the Assessment of Negative Symptoms, Subjective Experience of Negative Symptoms | N/A | Positive and negative schizophrenia symptoms | Not reported | Controls | -Under intoxication, participants reported significantly more negative symptoms than in placebo conditions |
| (Morrison et al., 2009) | UK male adults, (18-55); naïve cannabis users (N=22) | Randomized, double-blind, placebo-controlled, crossover design; 2.5mg THC or placebo; battery of clinical assessments, cognitive battery | N/A | Psychotic, anxious, and depressive symptoms, verbal learning and memory, attention, reasoning | N/A | Controls | -Intoxicated participants reported greater positive psychotic symptoms and anxious symptoms  -Intoxicated participants demonstrated impairments in working memory, episodic memory, attention, and reasoning |
| (Mustonen et al., 2018) | Finnish birth cohort; national representative sample (N=6534) | Prospective, birth cohort, longitudinal design (30yr follow-up); International Classification of Diseases -10th Version-Clinical Modification | 8 | Psychosis | Prodromal symptoms at 15-16, parental psychosis, substance use, socioeconomic status, family structure, place of residence, | N/A | -Adolescent cannabis use was a significant risk factor for psychosis at follow-up even after adjusting for possible covariates |
| (Otten & Engels, 2013) | Dutch adolescents at baseline (14-17); national representative sample (N=310) | Longitudinal cohort design (annual 4-yr follow-up); 6-item Depressive Mood List; Genotyping of 5‐HTTLPR | 7 | Depression Symptomology | Personality, alcohol and tobacco use, parental education level, parental practices | N/A | - Cannabis use increased the risk for depressive symptoms over time but only in the presence of the short allele of the 5‐HTTLPR genotype |
| (Pacheco-Colón et al., 2018) | US adolescents (14-18); frequent and infrequent cannabis users (N=79) | Cross-sectional, self-report; Apathy Evaluation Scale,  Motivation and Engagement Scale | N/A | Motivation | Sex, IQ, depression, anxiety, ADHD  symptoms, and amount and frequency of lifetime and past  30-day alcohol and nicotine use | Recreational and Chronic | -No differences in motivation emerged between groups |
| (Padula, Schweinsburg, & Tapert, 2007) | US adolescents (16-18); heavy cannabis users and non-users (N=34) | fMRI cross-sectional design; Beck Depression Inventory, Spielberger State Trait Anxiety Inventory; Spatial Working memory Task | 6 | Spatial working memory | Age, gender, ethnicity, family history of substance use disorders, depressive and anxious symptoms | Recreational, Chronic, and Dependent | -No behavior differences between groups emerged |
| (Patte, Qian, & Leatherdale, 2017) | Canadian adolescents at baseline (14-18); national representative sample (N=26475) | Longitudinal cohort design (annual 2yr follow-up); battery of clinical assessments | 7 | Academic aspirations, expectations, and performance, school engagement | Sex, grade, ethnicity, and tobacco use | N/A | -Frequent cannabis use at baseline was a significant risk factor for poor school engagement and academic performance at follow-up |
| (Patton et al., 2002) | Australian adolescents at baseline (14-15); national representative sample (N=1601) | Longitudinal cohort (annual 7-yr follow-up); Clinical interview schedule-revised, early delinquency scale | 7 | Anxiety, Depression | sex, age, rural or urban residence, parental education | N/A | - Daily cannabis use in girls was a significant risk factor for reporting depression and anxiety at follow-up  -Weekly cannabis use was a significant risk factor for reporting depression and anxiety at follow-up |
| (Pope Jr, Gruber, Hudson, Huestis, & Yurgelun‐Todd, 2002) | US adults (20 – 55); current heavy cannabis users, former heavy cannabis users, non-users (N=164) | Case-control design, neurocognitive battery | 9 | Executive function, attention, visuospatial memory, verbal memory | age, sex, ethnicity, mother's  and father's educational attainment, parental household income, and presence of substance abuse or psychiatric disorders in a first-degree relative | Chronic | -No behavior differences between groups emerged |
| (Pope et al., 2003) | US adults (18-65); adolescent-onset cannabis users, adult-onset cannabis users, non-users (N=209) | Cross-sectional design, 28-day cannabis abstinence; neurocognitive battery | 7 | Verbal memory, visuospatial memory, attention, executive function | Age, sex, ethnicity, education, SES | Chronic and Dependent | Adolescent-onset cannabis users demonstrated significant impairment in cognitive performance, especially in verbal IQ, in comparison to non-users and adult-onset cannabis users |
| (Rabin et al., 2017) | Canadian male adults (18-65); cannabis dependent patients with schizophrenia, non-clinical cannabis-dependent users | Repeated-measures design, 28-day cannabis abstinence (assessments at Day 0, 14, 28); neurocognitive battery, battery of battery of clinical assessments | 7 | Visuospatial working memory, working memory, executive functioning, sustained attention, psychomotor speed, verbal memory | Education level, IQ | Cannabis dependent | -Patients with schizophrenia demonstrated significant improvement in verbal memory by Day 28  -controls did not demonstrate significant improvement in any cognitive domain by Day 28 |
| (Ramaekers et al., 2006) | Dutch adults (19-29); recreational cannabis users (N=20) | Double-blind, placebo-controlled, two-way mixed model design; 0, 250, and 500 μg/kg THC; Neurocognitive battery | N/A | Motor control, executive function, motor impulsivity, |  | Recreational | -THC intoxication significantly impaired executive function and motor control in a dose-dependent fashion |
| (Ramaekers et al., 2009) | Dutch adults (18-55); occasional and heavy cannabis users (N=24) | double-blind, placebo-controlled, two-way mixed model design; placebo and 500 μg/kg THC; neurocognitive battery | N/A | Perceptual motor control, divided attention, motor inhibition, executive function | Cannabis use history | Recreational, Chronic, and Dependent | -Intoxication led to significant impairments in executive functioning in both groups  - Intoxication led to significant impairments in divided attention, motor control, and motor inhibition among occasional users  - Intoxication led to significant impairments in motor inhibition among heavy users |
| (Rössler, Hengartner, Angst, & Ajdacic‐Gross, 2012) | Swiss adolescents at baseline (19-20); national representative sample (N=591) | Prospective longitudinal design (follow-up at ages 21/22, 26/27, 28/29, 31/32, 37/38, and 46/47); Structured Psychopathological Interview, Social Consequences of Psychological Disturbances for Epidemiology | 8 | Schizophrenia and schizotypal symptomology, | Sex/gender, family background, socioeconomic status, family and school problems | N/A | -Regular cannabis use in adolescent was a significant risk factor for schizotypal symptomology at follow-up |
| (Ruiz-Veguilla et al., 2013) | Spanish adults (25-60); recreational cannabis users, daily users, and non-users (N= 185) | Cross-sectional, self-report design; Community Assessment of Psychic Experience | 7 | Psychotic symptomology | Sex, age, social exclusion, age of onset of cannabis use, other drug use | Recreational and Chronic | -Daily cannabis users reported more manic symptoms and auditory hallucinations than less frequent cannabis users  -Heavy cannabis users reported the most delusions |
| (Sagar et al., 2015) | US adolescents and adults (17-46); early-onset users, late-onset users, and non-users (N=84) | Cross-sectional design; Stroop Color Word Test | 9 | Executive function | -Age, IQ, alcohol use | Chronic and Dependent | -Early-onset users demonstrated impairment in executive function in comparison to non-users and late-onset users |
| (Schoeler et al., 2018) | UK male, boys at baseline (8); national representative sample (N=411) | Prospective longitudinal design (follow-up at ages 10, 14, 16, 18, 32, and 48); Structured Clinical Interview for DSM-IV | 7 | Depression | Alcohol, nicotine, and other drug use, SES, other psychiatric illness, behavior and emotional problems in childhood, childhood anxiety, and childhood conduct problems | N/A | -Early and frequent cannabis use before age 18 was a significant risk factor for depression at final follow-up |
| (Scott et al., 2017) | US adolescents and young adults (14-21); national representative sample of occasional and frequent cannabis users, non-users (N=4568) | Prospective, longitudinal design (findings only from T1); neurocognitive battery | 7 | Executive control, episodic memory, complex cognition, social cognition | Sex, age, neighborhood socioeconomic status, psychopathology, comorbid substance use | N/A | -Frequent cannabis users demonstrated impairments in executive control in comparison to occasional and non-users  -Younger age of first cannabis use was associated with greater impairment |
| (Shannon, Mathias, Dougherty, & Liguori, 2010) | US adolescents (12-17); low- and high-THC cannabis users (N=35) | Cross-sectional design; Iowa Gambling Task, Rapid Visual Processing | N/A | Decision-making, planning, sustained attention | Age, sex, IQ, | Chronic | -High THC cannabis users demonstrated greater impairments in decision-making and sustained attention than low THC users |
| (Smith, Longo, Fried, Hogan, & Cameron, 2010) | Canadian adolescents (19-21); cannabis users and non-users (N=24) | Cross-sectional design; visuospatial 2-back task | 9 | Visuospatial memory | Nicotine use, SES | Chronic | -No behavior differences between groups emerged |
| (Solowij, Michie, & Fox, 1995) | NZ adults (18-55); long-term, short-term-, heavy-, and light-cannabis users, non-users (N=80) | EEG randomized, controlled, counterbalanced, cross-sectional design; Auditory attention task | 6 | Selective attention | Age, education, sex, IQ | Recreational and Chronic | - No behavior differences between groups emerged |
| (Solowij et al., 2011) | Australian adolescents (16-20); cannabis users, alcohol users, and non-users (N=181) | Cross-sectional design; Rey Auditory Verbal Learning Test | 6 | Verbal learning, Memory | Age, education, IQ, alcohol consumption for cannabis and alcohol users | Chronic | -Cannabis users demonstrated significant memory impairments than alcohol users and non-users |
| (Stiby et al., 2015) | UK birth cohort; national representative sample (N=1155) | Longitudinal, birth cohort design (follow-up at ages 2, 7, 13, 15, 16, 18); Cannabis Abuse Screening Test | 9 | General Certificate of Secondary Education | maternal substance use, life course socio-economic position, sex, age, child educational attainment prior to age 11, child substance use, child conduct disorder | N/A | -Both cannabis and tobacco use at age 15 were associated with subsequent adverse educational outcomes by final follow-up |
| (Tapert et al., 2007) | US adolescents (16-18); cannabis users and non-users (N=43) | fMRI cross-sectional design; Go/no-go task | 6 | Response inhibition | Age, education, IQ | Chronic and Dependent | -No behavior differences between groups emerged |
| (Thames, Arbid, & Sayegh, 2014) | US adults (18-55); current cannabis users, past users, and non-users (N=158) | Cross-sectional design; Neurocognitive battery; urine toxicology | 6 | Attention, working memory, information processing speed, verbal learning, executive function | Education | Chronic | - Current users demonstrated impairment in attention and working memory in comparison to past- and non-users  - Current and past-users demonstrated impairment in executive function and information processing speed in comparison to non-users |
| (Thayer, YorkWilliams, Hutchison, & Bryan, 2019) | US older adults (60 – 80); current cannabis users and non-users (N = 56) | Cross-sectional design; Neurocognitive Battery | 7 | Impulsivity, working memory, executive function, processing speed, episodic memory | Depressive symptoms, age, anxious symptoms, tobacco use | Chronic and Dependent | -Current users demonstrated impairment in working memory in comparison to non=users  -No behavior differences in impulsivity, decision-making, processing speed, and episodic memory emerged |
| (Theunissen et al., 2012) | Dutch adults (18-55); occasional and heavy cannabis users (N=24) | EEG double-blind, placebo-controlled, two-way crossover design; 500 μg/kg body weight THC; divided attention task, stop signal task | N/A | Attention, inhibitory control | Not reported | Recreational, chronic, and dependent | -THC intoxication led to significant impairments in divided attention and inhibitory control |
| (Thompson, Leadbeater, Ames, & Merrin, 2019) | Canadian adolescents at baseline (12-18); national representative sample (N=662) | Prospective, longitudinal design (follow-up at years 2, 4, 6, 8, 10); Hollingshead Occupational Status Scale, self-report of educational attainment | 8 | Economic well-being, educational attainment | Sex, age, alcohol use, tobacco use, Oppositional defiant disorder, depressive and anxious symptomology | N/A | -Cannabis use was a risk factor for poorer educational and economic attainment in a dose-dependent manner |
| (Vaidya et al., 2012) | US Adults (18-55); frequent cannabis users and non-users (N=88) | Cross-sectional design; Iowa Gambling Task, | 8 | Decision-making | Education | Chronic and Dependent | - No behavior differences between groups emerged |
| (Van Laar, Van Dorsselaer, Monshouwer, & De Graaf, 2007) | Dutch adults at baseline (18-64); national representative sample (N=3854) | Longitudinal, cohort design (3yr follow-up); Composite International Diagnostic Interview | 9 | Psychiatric disorders | age, gender, education, urbanicity, parental health history, other substance use, childhood trauma, | N/A | -Cannabis use at baseline was a moderate risk factor for depression  -Cannabis use at baseline was a strong risk factor for bipolar disorder |
| (Van Os et al., 2002) | Dutch adults at baseline (18-64); national representative sample (N=4848) | Cohort, longitudinal design (1 and 3yr follow-up); Composite International Diagnostic Interview | 9 | Psychotic symptoms | Sex, race/ethnicity, education, unemployment, marital status, urbanicity, | N/A | -Cannabis use at baseline was a significant risk factor for psychosis and diagnosis of a psychotic disorder at follow-ups |
| (Verdejo-Garcia et al., 2007) | US male adults (25-55); cannabis users, cocaine users, and controls | Cross-sectional design; Iowa Gambling Task | 8 | Decision-making | education, maternal education, Shipley IQ, and Hollingshead Index of socioeconomic status, race, alcohol use, tobacco use | Chronic and Dependent | - Cannabis use was significantly related to impairments in decision-making in a dose-dependent manner  -Overall, Cannabis users performed the same as controls in the decision-making task however |
| (Wesley, Hanlon, & Porrino, 2011) | US adults (18-65); chronic cannabis users and non-users (N=32) | Cross-sectional; Iowa Gambling task | 8 | Decision-making | Anxiety and depressive symptomology | Chronic and Dependent | - Cannabis users demonstrated impairments in decision-making in comparison to non-users |
| (Wright, Scerpella, & Lisdahl, 2016) | US young adults (18-25); cannabis and non-users (N=84) | Cross-sectional, self-report design; battery of clinical assessments | 8 | Anxiety, depression, impulsivity, motivation, and executive dysfunction | Age, education, gender, race, | Recreational, Chronic, and Dependent | -Cannabis users reported decreased sensitivity to reward in comparison to non-users  -Cannabis users reported greater depressive and anxious symptomology than non-users, and the effect was especially pronounced in female users |
| (Zammit, Allebeck, Andreasson, Lundberg, & Lewis, 2002) | Swiss male adolescents at baseline (18-20) national representative sample (N= 50087) | Historical, cohort, longitudinal (27yr follow-up); Composite International Diagnostic Interview | 7 | Admissions to hospital for  ICD-8/9 schizophrenia and other psychoses | Disturbed childhood behaviours, alcohol misuse, family medical history, socioeconomic status, father’s occupation | N/A | -Cannabis use at baseline was a significant risk factor for development of schizophrenia in a dose-dependent manner |
| (Zuardi et al., 2017) | Brazilian adults (18-35); naïve cannabis users (N=60) | Randomized, double-blind, placebo-controlled, crossover design; placebo, 1mg clonazepam, 100-, 300-, or 900 mg CBD; Visual Analog Mood Scale, Test of Public Speaking in a Real Situation | N/A | Social Anxiety, affect | gender, age, BMI, | Controls | -Clonazepam and 300mg CBD significantly reduced anxiety scores in comparison to placebo and 100- or 900mg CBD |

*Table 2. Quality assessment of included case-control and cohort studies based on the Newcastle-Ottawa Scale (NOS).*

| (Author, Year) | Selection | | | | Comparability | | Outcome or Exposure | | | Total Points |
| --- | --- | --- | --- | --- | --- | --- | --- | --- | --- | --- |
|  | 1 | 2 | 3 | 4 | 1a* | 1b | 1** | 2 | 3 |  |
| (Arsenault et al., 2002) | **✓** | **✓** | **✓** | **✓** |  | **✓** | **✓** | **✓** | **✓** | **8** |
| (Assari, Mistry, Caldwell, & Zimmerman, 2018) | **✓** | **✓** | **✓** | **✓** |  | **✓** | **✓** | **✓** | **✓** | **8** |
| (Baggio et al., 2014) | **✓** | **✓** |  | **✓** | **✓** | **✓** |  |  | **✓** | **6** |
| (Bechtold et al., 2016) | **✓** | **✓** |  | **✓** | **✓** | **✓** |  | **✓** | **✓** | **7** |
| (Becker et al., 2018) | **✓** | **✓** | **✓** | **✓** | **✓** | **✓** | **✓** |  | **✓** | **8** |
| (Blanco et al., 2016) | **✓** | **✓** | **✓** |  |  | **✓** | **✓** | **✓** | **✓** | **7** |
| (Blest-Hopley et al., 2019) | **✓** | **✓** | **✓** | **✓** | **✓** | **✓** | **✓** | **✓** | **✓** | **9** |
| (Bloomfield, Morgan, Kapur, Curran, & Howes, 2014) | **✓** | **✓** | **✓** | **✓** |  | **✓** |  | **✓** | **✓** | **7** |
| (Boccio & Beaver, 2017) | **✓** | **✓** |  | **✓** |  | **✓** | **✓** | **✓** | **✓** | **7** |
| (Boden et al., 2020) | **✓** | **✓** | **✓** | **✓** | **✓** | **✓** | **✓** | **✓** | **✓** | **9** |
| (Bolla, Eldreth, Matochik, & Cadet, 2005) | **✓** | **✓** | **✓** | **✓** |  | **✓** | **✓** | **✓** | **✓** | **8** |
| (Burggren et al., 2018) | **✓** | **✓** | **✓** | **✓** | **✓** | **✓** | **✓** | **✓** | **✓** | **9** |
| (Carey et al., 2015) | **✓** | **✓** | **✓** | **✓** | **✓** | **✓** | **✓** | **✓** | **✓** | **9** |
| (Casey & Cservenka, 2020) | **✓** | **✓** | **✓** | **✓** |  | **✓** | **✓** | **✓** | **✓** | **8** |
| (Caspi et al., 2005) | **✓** | **✓** | **✓** | **✓** | **✓** | **✓** | **✓** | **✓** | **✓** | **9** |
| (Castellanos-Ryan et al., 2017) | **✓** | **✓** | **✓** | **✓** | **✓** | **✓** | **✓** | **✓** | **✓** | **9** |
| (Cousijn et al., 2014) |  | **✓** | **✓** | **✓** |  | **✓** |  | **✓** | **✓** | **6** |
| (Danielsson et al., 2016) | **✓** | **✓** |  |  | **✓** | **✓** |  | **✓** | **✓** | **6** |
| (Degenhardt et al., 2013) | **✓** | **✓** | **✓** | **✓** | **✓** | **✓** | **✓** | **✓** | **✓** | **9** |
| (Despina et al., 2020) | **✓** | **✓** | **✓** | **✓** | **✓** | **✓** | **✓** | **✓** | **✓** | **9** |
| (Dougherty et al., 2013) | **✓** | **✓** | **✓** | **✓** |  | **✓** | **✓** | **✓** | **✓** | **8** |
| (Duperrouzel et al., 2018) | **✓** | **✓** | **✓** |  | **✓** | **✓** |  |  | **✓** | **6** |
| (Epstein et al., 2015) | **✓** | **✓** |  |  |  | **✓** | **✓** | **✓** | **✓** | **6** |
| (Feingold et al., 2015) | **✓** | **✓** | **✓** |  | **✓** | **✓** | **✓** | **✓** | **✓** | **8** |
| (Feingold et al., 2016) | **✓** | **✓** | **✓** |  | **✓** | **✓** | **✓** | **✓** | **✓** | **8** |
| (Ferdinand et al., 2005) | **✓** | **✓** | **✓** | **✓** |  | **✓** | **✓** | **✓** | **✓** | **8** |
| (Fergusson et al., 2005) | **✓** | **✓** | **✓** | **✓** |  | **✓** | **✓** | **✓** | **✓** | **8** |
| (Fontes et al., 2011) | **✓** | **✓** | **✓** | **✓** |  | **✓** | **✓** | **✓** | **✓** | **8** |
| (Fridberg et al., 2013) | **✓** | **✓** | **✓** | **✓** |  | **✓** | **✓** | **✓** | **✓** | **8** |
| (Gage et al., 2014) | **✓** | **✓** |  | **✓** | **✓** | **✓** |  |  | **✓** | **6** |
| (Gage et al., 2015) | **✓** | **✓** |  | **✓** | **✓** | **✓** |  |  | **✓** | **6** |
| (Gonzalez et al., 2012) | **✓** | **✓** |  | **✓** |  | **✓** | **✓** | **✓** | **✓** | **8** |
| (Griffith-Lendering et al., 2013) | **✓** | **✓** |  | **✓** | **✓** | **✓** | **✓** | **✓** | **✓** | **8** |
| (MFH Griffith-Lendering et al., 2011) | **✓** | **✓** |  | **✓** | **✓** | **✓** | **✓** | **✓** | **✓** | **8** |
| (Gruber et al., 2012) | **✓** | **✓** | **✓** | **✓** |  | **✓** | **✓** | **✓** | **✓** | **8** |
| (Gruber et al., 2014) | **✓** | **✓** | **✓** | **✓** |  | **✓** | **✓** | **✓** | **✓** | **8** |
| (Grunberg et al., 2015) | **✓** | **✓** | **✓** | **✓** |  |  |  | **✓** | **✓** | **6** |
| (Harder et al., 2006) | **✓** | **✓** | **✓** |  | **✓** | **✓** |  | **✓** | **✓** | **7** |
| (Hatchard et al., 2014) |  | **✓** | **✓** | **✓** | **✓** | **✓** |  | **✓** | **✓** | **7** |
| (Hengartner et al., 2020) | **✓** | **✓** | **✓** | **✓** | **✓** | **✓** | **✓** | **✓** | **✓** | **9** |
| (Homel et al., 2014) | **✓** | **✓** | **✓** | **✓** |  | **✓** | **✓** | **✓** | **✓** | **8** |
| (Hayatbakhsh et al., 2007) | **✓** | **✓** | **✓** | **✓** | **✓** | **✓** |  | **✓** | **✓** | **8** |
| (Infante et al., 2020) | **✓** | **✓** | **✓** | **✓** | **✓** | **✓** | **✓** | **✓** | **✓** | **9** |
| (Jackson et al., 2016) | **✓** | **✓** | **✓** | **✓** |  | **✓** |  | **✓** | **✓** | **7** |
| (Jacobsen et al., 2004) | **✓** | **✓** | **✓** | **✓** |  | **✓** | **✓** | **✓** | **✓** | **8** |
| (Jager et al., 2010) | **✓** |  |  | **✓** | **✓** | **✓** | **✓** | **✓** | **✓** | **7** |
| (Jager et al., 2013) | **✓** |  |  | **✓** | **✓** | **✓** | **✓** | **✓** | **✓** | **7** |
| (Jager et al., 2006) | **✓** | **✓** | **✓** | **✓** | **✓** | **✓** | **✓** | **✓** | **✓** | **9** |
| (Kanayama et al., 2004) | **✓** | **✓** | **✓** | **✓** |  | **✓** | **✓** | **✓** | **✓** | **8** |
| (Kuepper et al., 2011) | **✓** | **✓** | **✓** |  |  | **✓** | **✓** | **✓** | **✓** | **7** |
| (Lane et al., 2005) | **✓** |  | **✓** | **✓** |  | **✓** | **✓** | **✓** | **✓** | **7** |
| (Lisdahl & Price, 2012) | **✓** | **✓** | **✓** | **✓** | **✓** | **✓** | **✓** | **✓** | **✓** | **9** |
| (Manrique-Garcia et al., 2012) |  | **✓** | **✓** |  | **✓** | **✓** | **✓** | **✓** | **✓** | **7** |
|  |  |  |  |  |  |  |  |  |  |  |
| (Martin-Soelch et al., 2009) |  | **✓** | **✓** | **✓** |  | **✓** |  | **✓** | **✓** | **6** |
| (Medina et al., 2007) | **✓** | **✓** | **✓** | **✓** | **✓** | **✓** | **✓** | **✓** | **✓** | **9** |
| (Meier et al., 2012) | **✓** | **✓** | **✓** | **✓** | **✓** | **✓** | **✓** | **✓** | **✓** | **9** |
| (Meier et al., 2015) |  | **✓** |  | **✓** |  | **✓** | **✓** | **✓** | **✓** | **6** |
| (Meier et al., 2018) | **✓** | **✓** | **✓** | **✓** |  | **✓** | **✓** | **✓** | **✓** | **8** |
| (Meier et al., 2020) | **✓** | **✓** |  |  | **✓** | **✓** |  | **✓** | **✓** | **6** |
| (Mercuri et al., 2018) |  | **✓** | **✓** | **✓** |  | **✓** |  | **✓** | **✓** | **6** |
| (Miettunen et al., 2008) | **✓** | **✓** |  | **✓** | **✓** | **✓** |  | **✓** | **✓** | **7** |
| (Mustonen et al., 2018) | **✓** | **✓** |  | **✓** | **✓** | **✓** | **✓** | **✓** | **✓** | **8** |
| (Otten & Engels, 2013) | **✓** | **✓** |  | **✓** | **✓** | **✓** |  | **✓** | **✓** | **7** |
| (Padula et al., 2007) |  | **✓** | **✓** | **✓** |  |  | **✓** | **✓** | **✓** | **6** |
| (Patte et al., 2017) | **✓** | **✓** |  | **✓** | **✓** | **✓** | **✓** |  | **✓** | **7** |
| (Patton et al., 2002) | **✓** | **✓** |  |  | **✓** | **✓** | **✓** | **✓** | **✓** | **7** |
| (Pope et al., 2002)) {, #727@@hidden}c | **✓** | **✓** | **✓** | **✓** | **✓** | **✓** | **✓** | **✓** | **✓** | **9** |
| (Pope et al., 2003) | **✓** | **✓** |  | **✓** |  | **✓** | **✓** | **✓** | **✓** | **7** |
| (Rabin et al., 2017) | **✓** |  | **✓** | **✓** |  | **✓** | **✓** | **✓** | **✓** | **7** |
| (Rössler et al., 2012) | **✓** | **✓** | **✓** | **✓** |  | **✓** | **✓** | **✓** | **✓** | **8** |
| (Ruiz-Veguilla et al., 2013) |  | **✓** | **✓** | **✓** | **✓** | **✓** |  | **✓** | **✓** | **7** |
| (Sagar et al., 2015) | **✓** | **✓** | **✓** | **✓** | **✓** | **✓** | **✓** | **✓** | **✓** | **9** |
| (Schoeler et al., 2018) |  | **✓** |  | **✓** | **✓** | **✓** | **✓** | **✓** | **✓** | **7** |
| (Scott et al., 2017) |  | **✓** | **✓** | **✓** | **✓** | **✓** |  | **✓** | **✓** | **7** |
| (Smith et al., 2010) | **✓** | **✓** | **✓** | **✓** | **✓** | **✓** | **✓** | **✓** | **✓** | **9** |
| (Solowij et al., 1995) |  | **✓** | **✓** | **✓** |  | **✓** |  | **✓** | **✓** | **6** |
| (Solowij et al., 2011) |  | **✓** | **✓** | **✓** |  | **✓** |  |  | **✓** | **6** |
| (Stiby et al., 2015) | **✓** | **✓** | **✓** | **✓** | **✓** | **✓** | **✓** | **✓** | **✓** | **9** |
| (Tapert et al., 2007) |  | **✓** | **✓** | **✓** |  | **✓** |  | **✓** | **✓** | **6** |
| (Thames et al., 2014) |  | **✓** | **✓** | **✓** |  | **✓** |  | **✓** | **✓** | **6** |
| (Thayer et al., 2019) |  | **✓** | **✓** | **✓** | **✓** | **✓** |  | **✓** | **✓** | **7** |
| (Thompson et al., 2019) | **✓** | **✓** |  | **✓** | **✓** | **✓** | **✓** | **✓** | **✓** | **8** |
| (Vaidya et al., 2012) | **✓** | **✓** | **✓** | **✓** |  | **✓** | **✓** | **✓** | **✓** | **8** |
| (Van Laar et al., 2007) | **✓** | **✓** | **✓** | **✓** | **✓** | **✓** | **✓** | **✓** | **✓** | **9** |
| (Van Os et al., 2002) | **✓** | **✓** | **✓** | **✓** | **✓** | **✓** | **✓** | **✓** | **✓** | **9** |
| (Verdejo-Garcia et al., 2007) | **✓** |  | **✓** | **✓** | **✓** | **✓** | **✓** | **✓** | **✓** | **8** |
| (Wesley et al., 2011) | **✓** | **✓** | **✓** | **✓** |  | **✓** | **✓** | **✓** | **✓** | **8** |
| (Wright et al., 2016) | **✓** | **✓** | **✓** | **✓** |  | **✓** | **✓** | **✓** | **✓** | **8** |
| (Zammit et al., 2002) |  | **✓** | **✓** | **✓** |  | **✓** | **✓** | **✓** | **✓** | **7** |

*Table 3. Quality assessment of excluded case-control and cohort studies based on the Newcastle-Ottawa Scale (NOS).*

| (Author, Year) | Selection | | | | Comparability | | Outcome or Exposure | | | Total Points |
| --- | --- | --- | --- | --- | --- | --- | --- | --- | --- | --- |
|  | 1 | 2 | 3 | 4 | 1a* | 1b | 1** | 2 | 3 |  |
| (Moreno et al., 2012) |  | **✓** | **✓** | **✓** |  |  |  | **✓** | **✓** | **5** |
| (Nusbaum et al., 2017) |  | **✓** |  | **✓** |  | **✓** |  | **✓** | **✓** | **5** |
| (Rasic, Weerasinghe, Asbridge, & Langille, 2013) | **✓** | **✓** |  |  | **✓** | **✓** |  | **✓** | **✓** | **5** |

**References**

Ansell, E. B., Laws, H. B., Roche, M. J., & Sinha, R. (2015). Effects of marijuana use on impulsivity and hostility in daily life. *Drug and alcohol dependence, 148*, 136-142.

Arkell, T. R., Lintzeris, N., Kevin, R. C., Ramaekers, J. G., Vandrey, R., Irwin, C., . . . McGregor, I. S. (2019). Cannabidiol (CBD) content in vaporized cannabis does not prevent tetrahydrocannabinol (THC)-induced impairment of driving and cognition. *Psychopharmacology, 236*(9), 2713-2724.

Arseneault, L., Cannon, M., Poulton, R., Murray, R., Caspi, A., & Moffitt, T. E. (2002). Cannabis use in adolescence and risk for adult psychosis: longitudinal prospective study. *BMJ, 325*(7374), 1212-1213.

Assari, S., Mistry, R., Caldwell, C. H., & Zimmerman, M. A. (2018). Marijuana use and depressive symptoms; gender differences in African American adolescents. *Frontiers in Psychology, 9*, 2135.

Atakan, Z., Bhattacharyya, S., Allen, P., Martin-Santos, R., Crippa, J., Borgwardt, S., . . . Stahl, D. (2013). Cannabis affects people differently: inter-subject variation in the psychotogenic effects of Δ9-tetrahydrocannabinol: a functional magnetic resonance imaging study with healthy volunteers. *Psychological medicine, 43*(6), 1255-1267.

Baggio, S., N'Goran, A. A., Deline, S., Studer, J., Dupuis, M., Henchoz, Y., . . . Gmel, G. (2014). Patterns of cannabis use and prospective associations with health issues among young males. *Addiction, 109*(6), 937-945. doi:10.1111/add.12490

Bechtold, J., Hipwell, A., Lewis, D. A., Loeber, R., & Pardini, D. (2016). Concurrent and sustained cumulative effects of adolescent marijuana use on subclinical psychotic symptoms. *American Journal of Psychiatry, 173*(8), 781-789.

Becker, B., Wagner, D., Gouzoulis-Mayfrank, E., Spuentrup, E., & Daumann, J. (2010). The impact of early-onset cannabis use on functional brain correlates of working memory. *Progress in Neuro-Psychopharmacology and Biological Psychiatry, 34*(6), 837-845.

Becker, M. P., Collins, P. F., Schultz, A., Urošević, S., Schmaling, B., & Luciana, M. (2018). Longitudinal changes in cognition in young adult cannabis users. *Journal of clinical and experimental neuropsychology, 40*(6), 529-543.

Bhattacharyya, S., Fusar-Poli, P., Borgwardt, S., Martin-Santos, R., Nosarti, C., O’Carroll, C., . . . Crippa, J. A. (2009). Modulation of mediotemporal and ventrostriatal function in humans by Δ9-tetrahydrocannabinol: a neural basis for the effects of Cannabis sativa on learning and psychosis. *Archives of general psychiatry, 66*(4), 442-451.

Blanco, C., Hasin, D. S., Wall, M. M., Flórez-Salamanca, L., Hoertel, N., Wang, S., . . . Olfson, M. (2016). Cannabis use and risk of psychiatric disorders: prospective evidence from a US national longitudinal study. *JAMA Psychiatry, 73*(4), 388-395.

Blest-Hopley, G., O’Neill, A., Wilson, R., Giampietro, V., & Bhattacharyya, S. (2019). Disrupted parahippocampal and midbrain function underlie slower verbal learning in adolescent-onset regular cannabis use. *Psychopharmacology*, 1-17.

Bloomfield, M. A., Morgan, C. J., Kapur, S., Curran, H. V., & Howes, O. D. (2014). The link between dopamine function and apathy in cannabis users: an [18 F]-DOPA PET imaging study. *Psychopharmacology, 231*(11), 2251-2259.

Boccio, C. M., & Beaver, K. M. (2017). Examining the influence of adolescent marijuana use on adult intelligence: Further evidence in the causation versus spuriousness debate. *Drug and alcohol dependence, 177*, 199-206.

Boden, J. M., Dhakal, B., Foulds, J. A., & Horwood, L. J. (2020). Life‐course trajectories of cannabis use: a latent class analysis of a New Zealand birth cohort. *Addiction, 115*(2), 279-290.

Boggs, D. L., Cortes-Briones, J. A., Surti, T., Luddy, C., Ranganathan, M., Cahill, J. D., . . . Skosnik, P. D. (2018). The dose-dependent psychomotor effects of intravenous delta-9-tetrahydrocannabinol (Δ9-THC) in humans. *Journal of Psychopharmacology, 32*(12), 1308-1318.

Bolla, K. I., Brown, K., Eldreth, D., Tate, K., & Cadet, J. L. (2002). Dose-related neurocognitive effects of marijuana use. *Neurology, 59*(9), 1337-1343. doi:10.1212/01.wnl.0000031422.66442.49

Bolla, K. I., Eldreth, D. A., Matochik, J. A., & Cadet, J. L. (2005). Neural substrates of faulty decision-making in abstinent marijuana users. *Neuroimage, 26*(2), 480-492.

Borgwardt, S. J., Allen, P., Bhattacharyya, S., Fusar-Poli, P., Crippa, J. A., Seal, M. L., . . . O'Carroll, C. (2008). Neural basis of Δ-9-tetrahydrocannabinol and cannabidiol: effects during response inhibition. *Biological psychiatry, 64*(11), 966-973.

Burggren, A. C., Siddarth, P., Mahmood, Z., London, E. D., Harrison, T. M., Merrill, D. A., . . . Bookheimer, S. Y. (2018). Subregional hippocampal thickness abnormalities in older adults with a history of heavy cannabis use. *Cannabis and cannabinoid research, 3*(1), 242-251.

Carey, S. E., Nestor, L., Jones, J., Garavan, H., & Hester, R. (2015). Impaired learning from errors in cannabis users: Dorsal anterior cingulate cortex and hippocampus hypoactivity. *Drug and alcohol dependence, 155*, 175-182.

Casey, J. L., & Cservenka, A. (2020). Effects of frequent marijuana use on risky decision-making in young adult college students. *Addictive behaviors reports*, 100253.

Caspi, A., Moffitt, T. E., Cannon, M., McClay, J., Murray, R., Harrington, H., . . . Braithwaite, A. (2005). Moderation of the effect of adolescent-onset cannabis use on adult psychosis by a functional polymorphism in the catechol-O-methyltransferase gene: longitudinal evidence of a gene X environment interaction. *Biological psychiatry, 57*(10), 1117-1127.

Castellanos-Ryan, N., Pingault, J.-B., Parent, S., Vitaro, F., Tremblay, R. E., & Seguin, J. R. (2017). Adolescent cannabis use, change in neurocognitive function, and high-school graduation: A longitudinal study from early adolescence to young adulthood. *Development and psychopathology, 29*(4), 1253.

Childs, E., Lutz, J. A., & de Wit, H. (2017). Dose-related effects of delta-9-THC on emotional responses to acute psychosocial stress. *Drug and alcohol dependence, 177*, 136-144.

Cousijn, J., Wiers, R. W., Ridderinkhof, K. R., van den Brink, W., Veltman, D. J., & Goudriaan, A. E. (2014). Effect of baseline cannabis use and working‐memory network function on changes in cannabis use in heavy cannabis users: a prospective fMRI study. *Human brain mapping, 35*(5), 2470-2482.

Curran, V. H., Brignell, C., Fletcher, S., Middleton, P., & Henry, J. (2002). Cognitive and subjective dose-response effects of acute oral Δ 9-tetrahydrocannabinol (THC) in infrequent cannabis users. *Psychopharmacology, 164*(1), 61-70.

D’Souza, D. C., Abi-Saab, W. M., Madonick, S., Forselius-Bielen, K., Doersch, A., Braley, G., . . . Krystal, J. H. (2005). Delta-9-tetrahydrocannabinol effects in schizophrenia: implications for cognition, psychosis, and addiction. *Biological psychiatry, 57*(6), 594-608.

Danielsson, A.-K., Lundin, A., Agardh, E., Allebeck, P., & Forsell, Y. (2016). Cannabis use, depression and anxiety: A 3-year prospective population-based study. *Journal of affective disorders, 193*, 103-108.

Degenhardt, L., Coffey, C., Romaniuk, H., Swift, W., Carlin, J. B., Hall, W. D., & Patton, G. C. (2013). The persistence of the association between adolescent cannabis use and common mental disorders into young adulthood. *Addiction, 108*(1), 124-133.

Despina, B., Massimiliano, O., Natalie, C.-R., Johanne, R., Tina, M., Michel, B., . . . Côté, M. S. (2020). Cannabis use, depression and suicidal ideation in adolescence: direction of associations in a population based cohort. *Journal of affective disorders*.

Desrosiers, N. A., Ramaekers, J. G., Chauchard, E., Gorelick, D. A., & Huestis, M. A. (2015). Smoked cannabis' psychomotor and neurocognitive effects in occasional and frequent smokers. *Journal of analytical toxicology, 39*(4), 251-261.

Dougherty, D. M., Mathias, C. W., Dawes, M. A., Furr, R. M., Charles, N. E., Liguori, A., . . . Acheson, A. (2013). Impulsivity, attention, memory, and decision-making among adolescent marijuana users. *Psychopharmacology, 226*(2), 307-319.

Duperrouzel, J., Hawes, S. W., Lopez-Quintero, C., Pacheco-Colón, I., Comer, J., & Gonzalez, R. (2018). The association between adolescent cannabis use and anxiety: a parallel process analysis. *Addictive Behaviors, 78*, 107-113.

Englund, A., Atakan, Z., Kralj, A., Tunstall, N., Murray, R., & Morrison, P. (2016). The effect of five day dosing with THCV on THC-induced cognitive, psychological and physiological effects in healthy male human volunteers: a placebo-controlled, double-blind, crossover pilot trial. *Journal of Psychopharmacology, 30*(2), 140-151.

Englund, A., Morrison, P. D., Nottage, J., Hague, D., Kane, F., Bonaccorso, S., . . . Holt, D. (2013). Cannabidiol inhibits THC-elicited paranoid symptoms and hippocampal-dependent memory impairment. *Journal of Psychopharmacology, 27*(1), 19-27.

Epstein, M., Hill, K. G., Nevell, A. M., Guttmannova, K., Bailey, J. A., Abbott, R. D., . . . Hawkins, J. D. (2015). Trajectories of marijuana use from adolescence into adulthood: environmental and individual correlates. *Developmental psychology, 51*(11), 1650.

Feingold, D., Weiser, M., Rehm, J., & Lev-Ran, S. (2015). The association between cannabis use and mood disorders: A longitudinal study. *J Affect Disord, 172*, 211-218. doi:10.1016/j.jad.2014.10.006

Feingold, D., Weiser, M., Rehm, J., & Lev-Ran, S. (2016). The association between cannabis use and anxiety disorders: results from a population-based representative sample. *European Neuropsychopharmacology, 26*(3), 493-505.

Ferdinand, R. F., Sondeijker, F., Van Der Ende, J., Selten, J. P., Huizink, A., & Verhulst, F. C. (2005). Cannabis use predicts future psychotic symptoms, and vice versa. *Addiction, 100*(5), 612-618.

Fergusson, D. M., Horwood, L. J., & Ridder, E. M. (2005). Tests of causal linkages between cannabis use and psychotic symptoms. *Addiction, 100*(3), 354-366.

Fontes, M. A., Bolla, K. I., Cunha, P. J., Almeida, P. P., Jungerman, F., Laranjeira, R. R., . . . Lacerda, A. L. (2011). Cannabis use before age 15 and subsequent executive functioning. *The British Journal of Psychiatry, 198*(6), 442-447.

Fridberg, D. J., Skosnik, P. D., Hetrick, W. P., & O’Donnell, B. F. (2013). Neural correlates of performance monitoring in chronic cannabis users and cannabis-naive controls. *Journal of Psychopharmacology, 27*(6), 515-525.

Gage, S., Hickman, M., Heron, J., Munafò, M., Lewis, G., Macleod, J., & Zammit, S. (2014). Associations of cannabis and cigarette use with psychotic experiences at age 18: findings from the Avon Longitudinal Study of Parents and Children. *Psychological medicine, 44*(16), 3435-3444.

Gage, S. H., Hickman, M., Heron, J., Munafò, M. R., Lewis, G., Macleod, J., & Zammit, S. (2015). Associations of cannabis and cigarette use with depression and anxiety at age 18: findings from the Avon Longitudinal Study of Parents and Children. *PloS one, 10*(4), e0122896.

Gonzalez, R., Schuster, R. M., Mermelstein, R. J., Vassileva, J., Martin, E. M., & Diviak, K. R. (2012). Performance of young adult cannabis users on neurocognitive measures of impulsive behavior and their relationship to symptoms of cannabis use disorders. *Journal of clinical and experimental neuropsychology, 34*(9), 962-976.

Griffith-Lendering, M., Huijbregts, S. C., Mooijaart, A., Vollebergh, W., & Swaab, H. (2011). Cannabis use and development of externalizing and internalizing behaviour problems in early adolescence: A TRAILS study. *Drug and alcohol dependence, 116*(1-3), 11-17.

Griffith-Lendering, M., Wigman, J., Prince van Leeuwen, A., Huijbregts, S., Huizink, A. C., Ormel, J., . . . Vollebergh, W. A. (2013). Cannabis use and vulnerability for psychosis in early adolescence–a TRAILS study. *Addiction, 108*(4), 733-740.

Gruber, S. A., Dahlgren, M. K., Sagar, K. A., Gönenc, A., & Killgore, W. D. (2012). Age of onset of marijuana use impacts inhibitory processing. *Neuroscience letters, 511*(2), 89-94.

Gruber, S. A., Dahlgren, M. K., Sagar, K. A., Gönenç, A., & Lukas, S. E. (2014). Worth the wait: effects of age of onset of marijuana use on white matter and impulsivity. *Psychopharmacology, 231*(8), 1455-1465.

Grunberg, V. A., Cordova, K. A., Bidwell, L., & Ito, T. A. (2015). Can marijuana make it better? Prospective effects of marijuana and temperament on risk for anxiety and depression. *Psychology of Addictive Behaviors, 29*(3), 590.

Harder, V. S., Morral, A. R., & Arkes, J. (2006). Marijuana use and depression among adults: Testing for causal associations. *Addiction, 101*(10), 1463-1472.

Hatchard, T., Fried, P., Hogan, M., Cameron, I., & Smith, A. (2014). Marijuana use impacts cognitive interference: an fMRI investigation in young adults performing the counting Stroop task. *Journal of Addiction Research & Therapy, 5*(4), 197-203.

Hayatbakhsh, M. R., Najman, J. M., Jamrozik, K., Mamun, A. A., Alati, R., & Bor, W. (2007). Cannabis and anxiety and depression in young adults: a large prospective study. *Journal of the American Academy of Child & Adolescent Psychiatry, 46*(3), 408-417.

Hengartner, M. P., Angst, J., Ajdacic-Gross, V., & Rössler, W. (2020). Cannabis use during adolescence and the occurrence of depression, suicidality and anxiety disorder across adulthood: Findings from a longitudinal cohort study over 30 years. *Journal of affective disorders*.

Homel, J., Thompson, K., & Leadbeater, B. (2014). Trajectories of marijuana use in youth ages 15–25: Implications for postsecondary education experiences. *Journal of studies on alcohol and drugs, 75*(4), 674-683.

Hunault, C. C., Böcker, K. B., Stellato, R., Kenemans, J. L., de Vries, I., & Meulenbelt, J. (2014). Acute subjective effects after smoking joints containing up to 69 mg Δ9-tetrahydrocannabinol in recreational users: a randomized, crossover clinical trial. *Psychopharmacology, 231*(24), 4723-4733.

Infante, M. A., Nguyen-Louie, T. T., Worley, M., Courtney, K. E., Coronado, C., & Jacobus, J. (2020). Neuropsychological trajectories associated with adolescent alcohol and cannabis use: A prospective 14-year study. *Journal of the International Neuropsychological Society, 26*(5), 480-491.

Jackson, N. J., Isen, J. D., Khoddam, R., Irons, D., Tuvblad, C., Iacono, W. G., . . . Baker, L. A. (2016). Impact of adolescent marijuana use on intelligence: Results from two longitudinal twin studies. *Proc Natl Acad Sci U S A, 113*(5), E500-508. doi:10.1073/pnas.1516648113

Jacobsen, L. K., Mencl, W. E., Westerveld, M., & Pugh, K. R. (2004). Impact of cannabis use on brain function in adolescents. *Ann N Y Acad Sci, 1021*, 384-390. doi:10.1196/annals.1308.053

Jager, G., Block, R. I., Luijten, M., & Ramsey, N. F. (2010). Cannabis use and memory brain function in adolescent boys: a cross-sectional multicenter functional magnetic resonance imaging study. *Journal of the American Academy of Child & Adolescent Psychiatry, 49*(6), 561-572. e563.

Jager, G., Block, R. I., Luijten, M., & Ramsey, N. F. (2013). Tentative evidence for striatal hyperactivity in adolescent cannabis-using boys: a cross-sectional multicenter fMRI study. *Journal of psychoactive drugs, 45*(2), 156-167.

Jager, G., Kahn, R. S., Van Den Brink, W., Van Ree, J. M., & Ramsey, N. F. (2006). Long-term effects of frequent cannabis use on working memory and attention: an fMRI study. *Psychopharmacology, 185*(3), 358-368.

Kanayama, G., Rogowska, J., Pope, H. G., Gruber, S. A., & Yurgelun-Todd, D. A. (2004). Spatial working memory in heavy cannabis users: a functional magnetic resonance imaging study. *Psychopharmacology, 176*(3-4), 239-247.

Kaufmann, R., Kraft, B., Frey, R., Winkler, D., Weiszenbichler, S., Bäcker, C., . . . Kress, H. (2010). Acute psychotropic effects of oral cannabis extract with a defined content of Δ9-tetrahydrocannabinol (THC) in healthy volunteers. *Pharmacopsychiatry, 43*(01), 24-32.

Kloft, L., Otgaar, H., Blokland, A., Monds, L. A., Toennes, S. W., Loftus, E. F., & Ramaekers, J. G. (2020). Cannabis increases susceptibility to false memory. *Proceedings of the National Academy of Sciences, 117*(9), 4585-4589.

Kuepper, R., van Os, J., Lieb, R., Wittchen, H.-U., Höfler, M., & Henquet, C. (2011). Continued cannabis use and risk of incidence and persistence of psychotic symptoms: 10 year follow-up cohort study. *BMJ, 342*.

Lane, S. D., Cherek, D. R., Pietras, C. J., & Steinberg, J. L. (2005). Performance of heavy marijuana-smoking adolescents on a laboratory measure of motivation. *Addictive Behaviors, 30*(4), 815-828.

Linares, I. M., Zuardi, A. W., Pereira, L. C., Queiroz, R. H., Mechoulam, R., Guimaraes, F. S., & Crippa, J. A. (2019). Cannabidiol presents an inverted U-shaped dose-response curve in a simulated public speaking test. *Brazilian Journal of Psychiatry, 41*(1), 9-14.

Lisdahl, K. M., & Price, J. S. (2012). Increased marijuana use and gender predict poorer cognitive functioning in adolescents and emerging adults. *J Int Neuropsychol Soc, 18*(4), 678-688. doi:10.1017/S1355617712000276

Lovell, M., Bruno, R., Johnston, J., Matthews, A., McGregor, I., Allsop, D., & Lintzeris, N. (2018). Cognitive, physical, and mental health outcomes between long-term cannabis and tobacco users. *Addictive Behaviors, 79*, 178-188.

Manrique-Garcia, E., Zammit, S., Dalman, C., Hemmingsson, T., & Allebeck, P. (2012). Cannabis use and depression: a longitudinal study of a national cohort of Swedish conscripts. *BMC Psychiatry, 12*(1), 112.

Martin-Santos, R., a Crippa, J., Batalla, A., Bhattacharyya, S., Atakan, Z., Borgwardt, S., . . . Farre, M. (2012). Acute effects of a single, oral dose of d9-tetrahydrocannabinol (THC) and cannabidiol (CBD) administration in healthy volunteers. *Current pharmaceutical design, 18*(32), 4966-4979.

Martin-Soelch, C., Kobel, M., Stoecklin, M., Michael, T., Weber, S., Krebs, B., & Opwis, K. (2009). Reduced response to reward in smokers and cannabis users. *Neuropsychobiology, 60*(2), 94-103.

Matheson, J., Sproule, B., Di Ciano, P., Fares, A., Le Foll, B., Mann, R. E., & Brands, B. (2020). Sex differences in the acute effects of smoked cannabis: evidence from a human laboratory study of young adults. *Psychopharmacology, 237*(2), 305-316.

McDonald, J., Schleifer, L., Richards, J. B., & de Wit, H. (2003). Effects of THC on behavioral measures of impulsivity in humans. *Neuropsychopharmacology, 28*(7), 1356-1365.

Medina, K. L., Hanson, K. L., Schweinsburg, A. D., Cohen-Zion, M., Nagel, B. J., & Tapert, S. F. (2007). Neuropsychological functioning in adolescent marijuana users: subtle deficits detectable after a month of abstinence. *Journal of the International Neuropsychological Society: JINS, 13*(5), 807.

Meier, M. H., Beardslee, J., & Pardini, D. (2020). Associations between recent and cumulative cannabis use and internalizing problems in boys from adolescence to young adulthood. *Journal of abnormal child psychology*, 1-12.

Meier, M. H., Caspi, A., Ambler, A., Harrington, H., Houts, R., Keefe, R. S., . . . Moffitt, T. E. (2012). Persistent cannabis users show neuropsychological decline from childhood to midlife. *Proc Natl Acad Sci U S A, 109*(40), E2657-2664. doi:10.1073/pnas.1206820109

Meier, M. H., Caspi, A., Danese, A., Fisher, H. L., Houts, R., Arseneault, L., & Moffitt, T. E. (2018). Associations between adolescent cannabis use and neuropsychological decline: a longitudinal co‐twin control study. *Addiction, 113*(2), 257-265.

Meier, M. H., Hill, M. L., Small, P. J., & Luthar, S. S. (2015). Associations of adolescent cannabis use with academic performance and mental health: a longitudinal study of upper middle class youth. *Drug and alcohol dependence, 156*, 207-212.

Mercuri, K., Terrett, G., Henry, J. D., Curran, H. V., Elliott, M., & Rendell, P. G. (2018). Episodic foresight deficits in regular, but not recreational, cannabis users. *Journal of Psychopharmacology, 32*(8), 876-882.

Miettunen, J., Törmänen, S., Murray, G. K., Jones, P. B., Mäki, P., Ebeling, H., . . . Joukamaa, M. (2008). Association of cannabis use with prodromal symptoms of psychosis in adolescence. *The British Journal of Psychiatry, 192*(6), 470-471.

Mokrysz, C., Freeman, T. P., Korkki, S., Griffiths, K., & Curran, H. V. (2016). Are adolescents more vulnerable to the harmful effects of cannabis than adults? A placebo-controlled study in human males. *Translational psychiatry, 6*(11), e961-e961.

Mokrysz, C., Landy, R., Gage, S. H., Munafo, M. R., Roiser, J. P., & Curran, H. V. (2016). Are IQ and educational outcomes in teenagers related to their cannabis use? A prospective cohort study. *Journal of Psychopharmacology, 30*(2), 159-168.

Mokrysz, C., Shaban, N. D., Freeman, T. P., Lawn, W., Pope, R. A., Hindocha, C., . . . Morgan, C. J. (2020). Acute effects of cannabis on speech illusions and psychotic-like symptoms: two studies testing the moderating effects of cannabidiol and adolescence. *Psychological medicine*, 1-9.

Moreno, M., Estevez, A. F., Zaldivar, F., Montes, J. M. G., Gutiérrez-Ferre, V. E., Esteban, L., . . . Flores, P. (2012). Impulsivity differences in recreational cannabis users and binge drinkers in a university population. *Drug and alcohol dependence, 124*(3), 355-362.

Morgan, C., Gardener, C., Schafer, G., Swan, S., Demarchi, C., Freeman, T., . . . Tan, N. (2012). Sub-chronic impact of cannabinoids in street cannabis on cognition, psychotic-like symptoms and psychological well-being. *Psychological medicine, 42*(2), 391.

Morgan, C. J., Schafer, G., Freeman, T. P., & Curran, H. V. (2010). Impact of cannabidiol on the acute memory and psychotomimetic effects of smoked cannabis: naturalistic study. *The British Journal of Psychiatry, 197*(4), 285-290.

Morrison, P., & Stone, J. (2011). Synthetic delta‐9‐tetrahydrocannabinol elicits schizophrenia‐like negative symptoms which are distinct from sedation. *Human Psychopharmacology: Clinical and Experimental, 26*(1), 77-80.

Morrison, P., Zois, V., McKeown, D., Lee, T., Holt, D., Powell, J., . . . Murray, R. (2009). The acute effects of synthetic intravenous [Delta] 9-tetrahydrocannabinol on psychosis, mood and cognitive functioning. *Psychological medicine, 39*(10), 1607.

Mustonen, A., Niemelä, S., Nordström, T., Murray, G. K., Mäki, P., Jääskeläinen, E., & Miettunen, J. (2018). Adolescent cannabis use, baseline prodromal symptoms and the risk of psychosis. *The British Journal of Psychiatry, 212*(4), 227-233.

Nusbaum, A. T., Whitney, P., Cuttler, C., Spradlin, A., Hinson, J. M., & McLaughlin, R. J. (2017). Altered attentional control strategies but spared executive functioning in chronic cannabis users. *Drug and alcohol dependence, 181*, 116-123.

Otten, R., & Engels, R. C. (2013). Testing bidirectional effects between cannabis use and depressive symptoms: moderation by the serotonin transporter gene. *Addiction biology, 18*(5), 826-835.

Pacheco-Colón, I., Coxe, S., Musser, E. D., Duperrouzel, J. C., Ross, J. M., & Gonzalez, R. (2018). Is Cannabis Use Associated with Various Indices of Motivation among Adolescents? *Substance use & misuse, 53*(7), 1158-1169.

Padula, C. B., Schweinsburg, A. D., & Tapert, S. F. (2007). Spatial working memory performance and fMRI activation interaction in abstinent adolescent marijuana users. *Psychology of Addictive Behaviors, 21*(4), 478.

Patte, K. A., Qian, W., & Leatherdale, S. T. (2017). Marijuana and alcohol use as predictors of academic achievement: a longitudinal analysis among youth in the COMPASS study. *Journal of school health, 87*(5), 310-318.

Patton, G. C., Coffey, C., Carlin, J. B., Degenhardt, L., Lynskey, M., & Hall, W. (2002). Cannabis use and mental health in young people: cohort study. *BMJ, 325*(7374), 1195-1198.

Pope Jr, H. G., Gruber, A. J., Hudson, J. I., Cohane, G., Huestis, M. A., & Yurgelun-Todd, D. (2003). Early-onset cannabis use and cognitive deficits: what is the nature of the association? *Drug and alcohol dependence, 69*(3), 303-310.

Pope Jr, H. G., Gruber, A. J., Hudson, J. I., Huestis, M. A., & Yurgelun‐Todd, D. (2002). Cognitive Measures in long‐term cannabis users. *The Journal of Clinical Pharmacology, 42*(S1), 41S-47S.

Rabin, R. A., Barr, M. S., Goodman, M. S., Herman, Y., Zakzanis, K. K., Kish, S. J., . . . George, T. P. (2017). Effects of Extended Cannabis Abstinence on Cognitive Outcomes in Cannabis Dependent Patients with Schizophrenia vs Non-Psychiatric Controls. *Neuropsychopharmacology, 42*(11), 2259-2271. doi:10.1038/npp.2017.85

Ramaekers, J. G., Kauert, G., Theunissen, E., Toennes, S. W., & Moeller, M. (2009). Neurocognitive performance during acute THC intoxication in heavy and occasional cannabis users. *Journal of Psychopharmacology, 23*(3), 266-277.

Ramaekers, J. G., Kauert, G., van Ruitenbeek, P., Theunissen, E. L., Schneider, E., & Moeller, M. R. (2006). High-potency marijuana impairs executive function and inhibitory motor control. *Neuropsychopharmacology, 31*(10), 2296-2303.

Rasic, D., Weerasinghe, S., Asbridge, M., & Langille, D. B. (2013). Longitudinal associations of cannabis and illicit drug use with depression, suicidal ideation and suicidal attempts among Nova Scotia high school students. *Drug and alcohol dependence, 129*(1-2), 49-53.

Rössler, W., Hengartner, M. P., Angst, J., & Ajdacic‐Gross, V. (2012). Linking substance use with symptoms of subclinical psychosis in a community cohort over 30 years. *Addiction, 107*(6), 1174-1184.

Ruiz-Veguilla, M., Barrigón, M. L., Hernández, L., Rubio, J. L., Gurpegui, M., Sarramea, F., . . . Ferrin, M. (2013). Dose–response effect between cannabis use and psychosis liability in a non-clinical population: evidence from a snowball sample. *Journal of psychiatric research, 47*(8), 1036-1043.

Sagar, K. A., Dahlgren, M. K., Gönenç, A., Racine, M. T., Dreman, M. W., & Gruber, S. A. (2015). The impact of initiation: Early onset marijuana smokers demonstrate altered Stroop performance and brain activation. *Developmental cognitive neuroscience, 16*, 84-92.

Schoeler, T., Theobald, D., Pingault, J.-B., Farrington, D., Coid, J., & Bhattacharyya, S. (2018). Developmental sensitivity to cannabis use patterns and risk for major depressive disorder in mid-life: findings from 40 years of follow-up. *Psychological medicine, 48*(13), 2169-2176.

Scott, J. C., Wolf, D. H., Calkins, M. E., Bach, E. C., Weidner, J., Ruparel, K., . . . Gur, R. E. (2017). Cognitive functioning of adolescent and young adult cannabis users in the Philadelphia Neurodevelopmental Cohort. *Psychology of Addictive Behaviors, 31*(4), 423.

Shannon, E. E., Mathias, C. W., Dougherty, D. M., & Liguori, A. (2010). Cognitive impairments in adolescent cannabis users are related to THC levels. *Addictive Disorders & Their Treatment, 9*(4), 158-163.

Smith, A. M., Longo, C. A., Fried, P. A., Hogan, M. J., & Cameron, I. (2010). Effects of marijuana on visuospatial working memory: an fMRI study in young adults. *Psychopharmacology, 210*(3), 429-438.

Solowij, N., Jones, K. A., Rozman, M. E., Davis, S. M., Ciarrochi, J., Heaven, P. C., . . . Yücel, M. (2011). Verbal learning and memory in adolescent cannabis users, alcohol users and non-users. *Psychopharmacology, 216*(1), 131-144.

Solowij, N., Michie, P. T., & Fox, A. M. (1995). Differential impairments of selective attention due to frequency and duration of cannabis use. *Biological psychiatry, 37*(10), 731-739.

Stiby, A. I., Hickman, M., Munafò, M. R., Heron, J., Yip, V. L., & Macleod, J. (2015). Adolescent cannabis and tobacco use and educational outcomes at age 16: birth cohort study. *Addiction, 110*(4), 658-668.

Tapert, S. F., Schweinsburg, A. D., Drummond, S. P., Paulus, M. P., Brown, S. A., Yang, T. T., & Frank, L. R. (2007). Functional MRI of inhibitory processing in abstinent adolescent marijuana users. *Psychopharmacology, 194*(2), 173-183.

Thames, A. D., Arbid, N., & Sayegh, P. (2014). Cannabis use and neurocognitive functioning in a non-clinical sample of users. *Addictive Behaviors, 39*(5), 994-999.

Thayer, R. E., YorkWilliams, S. L., Hutchison, K. E., & Bryan, A. D. (2019). Preliminary results from a pilot study examining brain structure in older adult cannabis users and nonusers. *Psychiatry Research: Neuroimaging, 285*, 58-63.

Theunissen, E. L., Kauert, G. F., Toennes, S. W., Moeller, M. R., Sambeth, A., Blanchard, M. M., & Ramaekers, J. G. (2012). Neurophysiological functioning of occasional and heavy cannabis users during THC intoxication. *Psychopharmacology, 220*(2), 341-350.

Thompson, K., Leadbeater, B., Ames, M., & Merrin, G. J. (2019). Associations between marijuana use trajectories and educational and occupational success in young adulthood. *Prevention science, 20*(2), 257-269.

Vaidya, J. G., Block, R. I., O'leary, D. S., Ponto, L. B., Ghoneim, M. M., & Bechara, A. (2012). Effects of chronic marijuana use on brain activity during monetary decision-making. *Neuropsychopharmacology, 37*(3), 618-629.

Van Laar, M., Van Dorsselaer, S., Monshouwer, K., & De Graaf, R. (2007). Does cannabis use predict the first incidence of mood and anxiety disorders in the adult population? *Addiction, 102*(8), 1251-1260.

Van Os, J., Bak, M., Hanssen, M., Bijl, R., De Graaf, R., & Verdoux, H. (2002). Cannabis use and psychosis: a longitudinal population-based study. *American journal of epidemiology, 156*(4), 319-327.

Verdejo-Garcia, A., Benbrook, A., Funderburk, F., David, P., Cadet, J.-L., & Bolla, K. I. (2007). The differential relationship between cocaine use and marijuana use on decision-making performance over repeat testing with the Iowa Gambling Task. *Drug and alcohol dependence, 90*(1), 2-11.

Wesley, M. J., Hanlon, C. A., & Porrino, L. J. (2011). Poor decision-making by chronic marijuana users is associated with decreased functional responsiveness to negative consequences. *Psychiatry Research: Neuroimaging, 191*(1), 51-59.

Wright, N. E., Scerpella, D., & Lisdahl, K. M. (2016). Marijuana use is associated with behavioral approach and depressive symptoms in adolescents and emerging adults. *PloS one, 11*(11), e0166005.

Zammit, S., Allebeck, P., Andreasson, S., Lundberg, I., & Lewis, G. (2002). Self reported cannabis use as a risk factor for schizophrenia in Swedish conscripts of 1969: historical cohort study. *BMJ, 325*(7374), 1199.

Zuardi, A. W., Rodrigues, N. P., Silva, A. L., Bernardo, S. A., Hallak, J. E., Guimarães, F. S., & Crippa, J. A. (2017). Inverted U-shaped dose-response curve of the anxiolytic effect of cannabidiol during public speaking in real life. *Frontiers in pharmacology, 8*, 259.
